# Mobile Apps That Promote Emotion Regulation, Positive Mental Health, and Well-being in the General Population: Systematic Review and Meta-analysis

**DOI:** 10.2196/31170

**Published:** 2021-11-08

**Authors:** Mia Eisenstadt, Shaun Liverpool, Elisa Infanti, Roberta Maria Ciuvat, Courtney Carlsson

**Affiliations:** 1 Evidence Based Practice Unit Anna Freud National Centre for Children and Families and University College London London United Kingdom; 2 Paradym Ltd Bloomsbury London United Kingdom; 3 Faculty of Health, Social Care and Medicine Edge Hill University Ormskirk United Kingdom; 4 Division of Psychology and Language Sciences University College London London United Kingdom; 5 Birkbeck Department of Psychological Sciences University of London London United Kingdom

**Keywords:** systematic review, MHapp, mHealth, mental health, well-being, emotion regulation, mobile apps, effectiveness, monitoring, management, mental health app

## Abstract

**Background:**

Among the general public, there appears to be a growing need and interest in receiving digital mental health and well-being support. In response to this, mental health apps (MHapps) are becoming available for monitoring, managing, and promoting positive mental health and well-being. Thus far, evidence supports favorable outcomes when users engage with MHapps, yet there is a relative paucity of reviews on apps that support positive mental health and well-being.

**Objective:**

We aimed to systematically review the available research on MHapps that promote emotion regulation, positive mental health, and well-being in the general population aged 18-45 years. More specifically, the review aimed at providing a systematic description of the theoretical background and features of MHapps while evaluating any potential effectiveness.

**Methods:**

A comprehensive literature search of key databases, including MEDLINE (via Ovid), EMBASE (via Ovid), PsycINFO (via Ovid), Web of Science, and the Cochrane Register of Controlled Trials (CENTRAL), was performed until January 2021. Studies were included if they described standalone mental health and well-being apps for adults without a formal mental health diagnosis. The quality of all studies was assessed against the Mixed Methods Appraisal Tool. In addition, the Cochrane Risk-of-Bias tool (RoB-2) was used to assess randomized control trials (RCTs). Data were extracted using a modified extraction form from the Cochrane Handbook of Systematic Reviews. A narrative synthesis and meta-analysis were then undertaken to address the review aims.

**Results:**

In total, 3156 abstracts were identified. Of these, 52 publications describing 48 MHapps met the inclusion criteria. Together, the studies evaluated interventions across 15 countries. Thirty-nine RCTs were identified suggesting some support for the role of individual MHapps in improving and promoting mental health and well-being. Regarding the pooled effect, MHapps, when compared to controls, showed a small effect for reducing mental health symptoms (*k*=19, Hedges *g*=–0.24, 95% CI –0.34 to –0.14; *P*<.001) and improving well-being (*k*=13, *g*=0.17, 95% CI 0.05-0.29, *P=*.004), and a medium effect for emotion regulation (*k*=6, *g*=0.49, 95% CI 0.23-0.74, *P*<.001). There is also a wide knowledge base of creative and innovative ways to engage users in techniques such as mood monitoring and guided exercises. Studies were generally assessed to contribute unclear or a high risk of bias, or to be of medium to low methodological quality.

**Conclusions:**

The emerging evidence for MHapps that promote positive mental health and well-being suggests promising outcomes. Despite a wide range of MHapps, few apps specifically promote emotion regulation. However, our findings may position emotion regulation as an important mechanism for inclusion in future MHapps. A fair proportion of the included studies were pilot or feasibility trials (*k*=17, 33%), and full-scale RCTs reported high attrition rates and nondiverse samples. Given the number and pace at which MHapps are being released, further robust research is warranted to inform the development and testing of evidence-based programs.

## Introduction

### Background

Globally, the prevalence of mental health disorders has been increasing [1]. Statistics from the United Kingdom indicate that between 16% and 21% of working adults experience mental health difficulties [2]. In 2019, a study found that there were 51.5 million North American adults with mental illness, with the highest prevalence among young adults aged 18-25 years (29.4%) compared to adults aged 26-49 (25%) or 50 years and older (14.1%) [3]. In light of these statistics, neuropsychiatric conditions continue to be among the main causes of disability globally [1]. In recent times, unfortunate events such as the COVID-19 pandemic have further exacerbated the problem, contributing to rising rates of anxiety, loneliness, and depression [1,4,5], along with decreased access to in-person support [6]. In this context, there is an increased demand for accessible and scalable mental health services (eg, mobile health and electronic health) for both the promotion of well-being and the prevention of mental disorders [7].

### Potential of Mental Health Apps to Fill the Need

Delivering mental health care online has become more feasible with the rapid increase in smartphone usage. Smartphone ownership is estimated to reach over 6 billion users globally in 2021 and these numbers are expected to increase by several hundred million in the coming few years [8]. Moreover, research suggests that in August 2017, smartphone owners in the United Kingdom spent on average 62 hours per month using the internet, as compared with 75 hours in the United States and 58 hours in Germany [9]. In general, digital apps may offer users opportunities to manage their mental and physical health, and support behavior change efforts. An estimate suggested that nearly 325,000 health apps were available for users to download in 2017, with mental health apps (MHapps) constituting about one-third of disease-specific apps [10,11]. In addition, a report from Statista found that “health and lifestyle” was one of the most popular categories of apps in the App Store [12]. However, the vast majority of MHapps have not been scientifically tested [13].

The developing evidence base for MHapps suggests that apps accessed via smart devices are increasingly able to play an important role in mental health care provision [13,14]. MHapps have been researched in terms of their effectiveness for the treatment and management of mental health disorders, but they are also increasingly understood to have potential in the prevention of mental health disorders and in the promotion of positive mental health [13-15]. In particular, the dynamic multifeatured nature of MHapps provides a platform for monitoring [16], preventing [14], and reducing mental health symptoms [17]. In addition, MHapps are able to facilitate emotion regulation and enhance mental well-being [14]. Moreover, MHapps are appealing to users due to advantages of cost-effectiveness, privacy, personalization features, and scope for use at any time in any location and setting [18]. MHapps also have the potential to overcome barriers to seeking help, such as stigma, as well as to promote positive habits for improved long-term well-being and mental health outcomes [14,18]. However, scientific research has not been able to keep up with the pace of the new developments in MHapps. Consequently, a large number of apps are available without any published scientific evidence base or peer-reviewed acceptability studies [19,20].

### Mental Well-being, Positive Mental Health, and Emotion Regulation

Although enhanced psychological well-being has been consistently linked to positive health and mental health outcomes, it is increasingly understood that mental health and mental well-being are separate entities with separate determinants [21,22]. The concept of mental well-being goes beyond the absence of mental health disorders and symptoms, and can address psychological parameters such as subjective well-being, autonomy, positive relationships, and personal growth [23,24]. In the same vein, positive mental health refers to “a positive emotion (affect) such as feelings of happiness, a personality trait inclusive of the psychological resources of self-esteem and mastery, and as resilience, which is the capacity to cope with adversity” [25]. Thus, both concepts overlap in highlighting that well-being is a broader concept that goes beyond the absence of mental health disorders [21]. Further, emotion regulation has been considered to be a focal point to address psychological disorders [26] and to enhance well-being [27]. Emotion regulation refers to the experience and expression of both positive and negative emotions [28,29]. Difficulties with emotion regulation are linked with increased stress [28,29], and represent an established risk factor for a range of mental health disorders such as depression [26] and bipolar disorder [30]. Emotion regulation strategies can be used in both adaptive and maladaptive ways depending on the context and the purpose. However, frequent use of maladaptive emotion regulation strategies is linked to mood disorders [30]. Therefore, being mindful or having emotional awareness is considered to facilitate emotion regulation [31], and may be considered an underlying influencing factor to achieve positive mental health and well-being [32].

### Previous Research and Systematic Reviews

Interestingly, despite the large number of apps available, the evidence of their effectiveness is not yet widely accepted. In a review of 52 commercially available anxiety apps, the authors reported that 67.3% did not include health care professionals in their creation and only 3.8% were supported by robust research [19]. Nonetheless, the growing evidence base suggests the potential efficacy of MHapps [33-35]. For example, Firth et al [34] found app-delivered interventions to be effective in decreasing anxiety (Hedges *g*=0.32, 95% CI 0.17-0.48) and depression (*g*=0.38, 95% CI 0.24-0.52) [34]. Similarly, other studies reported small to moderate effect sizes, specifically for mindfulness apps that reduced perceived stress (*g*=0.46, 95% CI 0.24-0.68), anxiety (*g*=0.28, 95% CI 0.16-0.40), and depression (*g*=0.33, 95% CI 0.24-0.43), and increased psychological well-being (*g*=0.29, 95% CI 0.14-0.45) [35]. Other reviewers corroborated this research, reporting positive findings for mindfulness apps in improving overall mental health (*g*=0.23, 95% CI 0.09-0.38) [36] and reducing perceived stress (*g*=–0.43, 95% CI –0.20 to –0.66) [37]. Other findings indicated some support for MHapps targeting alcohol disorder, sleep disorder, depression, suicidal behaviors, self-injurious thoughts/behaviors, and posttraumatic stress disorder (PTSD) [16,17]. Although this knowledge base points in a positive direction, there has been a stronger emphasis on the effectiveness of MHapps for monitoring and managing mental health disorders [16,17,33], cognitive behavioral therapy (CBT)-based MHapps, and MHapps tested within specific clinical populations [16,34,38]. With respect to evidence of the effectiveness of MHapps for the general public, McKay and colleagues [39] reviewed commercially available healthy lifestyle apps, and found that behavior change strategies mainly focused on rehearsal or practice (of new habits) and self-monitoring. A recent meta-analysis found a small effect of mindfulness MHapps for psychological well-being but found no significant effects for general well-being [35]. Building on these findings could provide support for MHapps that are underpinned by other psychological theories and highlight benefits for a broader sample of users.

### Rationale for This Review

Based on the above evidence, prior reviews examining the evidence for the effectiveness of MHapps did not include emotion regulation and rarely focused on mental well-being apps [14,20,40]. However, it is increasingly recognized that MHapps can support emotion regulation and may offer an advantage for users to manage their emotional states [14]. Moreover, the effectiveness of an intervention is usually associated with the level of user engagement [41], and therefore more research highlighting the components and features of the interface and design of MHapps would be beneficial. Although there are studies emerging that provide some recommendations of features that could be included in MHapps [14], it is still unclear how these features are being incorporated and the dominant theoretical approaches applied to the design. In this review, we were particularly interested in adults (18-45 years) owing to the concerning prevalence data indicating that among US adults aged 18 years or older, less than half of the population with a mental health disorder accessed mental health services (44.8% of adults >18 years old in 2019) [3]. With smartphone ownership being the greatest among young to middle-aged adults (91%-100% aged 18-44 years), and the evidence that this age group is the most likely to access and engage with smartphone apps [8], any important findings from this review may be readily transferable.

Thus, the overall objective of this review was to provide an overview of the available evidence on MHapps that promote emotion regulation, positive mental health, and well-being in the general adult population. This review will complement and expand upon the existing systematic reviews that have focused on apps for mental health disorders by focusing on MHapps for mental well-being and positive mental health. We aimed to identify, evaluate, and summarize the findings of relevant individual studies, thereby making the available evidence more accessible to both researchers and commercially based developers. More specifically, we describe and assess the characteristics and theoretical background of the apps themselves and the studies undertaken to evaluate them. We then highlight any gaps in the current knowledge base that may require further investigation. In doing so, we aimed to address the following research questions: (1) What are the characteristics and theoretical background of MHapps designed to improve (a) mental well-being (eg, psychological, subjective, and emotional), (b) emotion regulation (eg, emotion awareness), and (c) positive mental health (eg, reduce early mental health symptoms)? (2) Is there potential for MHapps to be effective in improving emotion regulation, positive mental health, and well-being in the general population (18-45 years)?

For the purpose of this study, an MHapp was defined as a digital psychological intervention or program that can be directly downloaded onto a mobile device. MHapps aim to promote positive mental health and well-being, including a reduction in mental health symptoms such as stress, and anxiety and depression symptomology. These apps are expected to be standalone interventions serving as a form of psychological intervention by assisting the user to draw on their own capacities to facilitate behavior change, and increase psychoeducation and self-help provisions [42,43].

## Methods

### Design

The review was performed in accordance with the Cochrane Handbook for Systematic Reviews of Interventions [44] and is reported according to the PRISMA (Preferred Reporting Items for Systematic Reviews and Meta-analyses) guidelines [45]. This study protocol was registered in the International Prospective Register of Systematic Reviews of the National Institute for Health Research (PROSPERO) [46] (ID CRD42020213051).

### Changes to the Review Protocol

Notably, changes were made after publication of the study protocol. We initially planned to only focus on studies that examined emotion regulation and well-being; however, we also ultimately included studies that measured positive mental health–related outcomes. This decision was based on the fact that many papers tend to interchange terms related to mental well-being and positive mental health [35]. Owing to the limited qualitative data found in the review, we did not summarize the results of qualitative studies as per the review protocol. As the review retrieved a large number of eligible studies that included outcomes of interest, we did not examine secondary outcomes as stated previously (ie, physical health and behavioral outcomes such as improved sleep). Such outcomes would be appropriately studied in a separate review and meta-analysis. Other changes included the use of the Mixed Methods Appraisal Tool (MMAT) instead of ROBINS-I to perform the quality assessment of the identified studies. MMAT was selected as it provided a single tool to assess methodological quality criteria for different study designs [47]. We initially intended to perform subgroup analysis based on age, gender, and ethnicity of the samples or the underpinning theory of the MHapp; however, owing to the high levels of heterogeneity, we were unable to perform these analyses. Therefore, we instead captured some key differences in the narrative synthesis (eg, the overrepresentation of groups with specific demographic characteristics).

### Search Strategy

A systematic literature search was completed using the following 5 electronic databases: MEDLINE (via Ovid), EMBASE (via Ovid), PsycINFO (via Ovid), Web of Science, and Cochrane Register of Controlled Trials (CENTRAL). The key terms for the intervention type (mobile apps) and the outcome themes (well-being OR emotion regulation OR mental health) were searched in the title, abstract, keywords, and, when available, subject headings. In addition, study type terms (including randomized controlled trials [RCTs] and before-and-after studies) were searched in the full text. Queries for each key area were combined with a logical “AND” operator, and were adapted to the different syntax and technical support of each individual database (see Multimedia Appendix 1). We aimed to identify records matching our inclusion criteria that were published between January 2008 and January 2021. We selected 2008 as the starting point in correspondence with the year when apps were first available to download on smart devices [33]. In addition, the bibliography of the relevant reviews and included studies were manually searched to identify additional publications for inclusion.

### Eligibility Criteria

We included (a) both qualitative and quantitative experimental (eg, any type of RCT, controlled before-after) or quasiexperimental (eg, one-group pretest-posttest design, time-series) studies; (b) studies investigating the effects of standalone psychological interventions focused on promoting the outcomes of psychological, mental, or emotional well-being, promoting emotion regulation and positive mental health; (c) studies in which interventions were delivered via a digital app accessed via smartphones or tablets, or other portable devices. In addition, studies were included if they were (d) targeting adults in the age range of 18-45 years or interventions that partially overlapped with the target population where the mean age of the participants fell between 18 and 45 years. Moreover, only (e) peer-reviewed studies and (f) those published in English were considered.

We excluded records focusing on a physical characteristic (eg, weight loss, physical activity, tracking alcohol consumption) as a primary outcome or those focusing on diagnosis or assessment only. Studies reporting digital interventions delivered in conjunction with in-person interventions or that focused on the evaluation of in-person therapies including a digital component or online services for scheduling/booking appointments were also excluded. Similarly, telehealth interventions such as therapy delivered by phone, text message, video platform, or personal computer were excluded. Owing to the focus of this study on the general population, we also excluded records focusing on diagnosed health disorders (eg, PTSD, schizophrenia, major depression) and neurodiverse conditions (eg, dyslexia and autism spectrum disorders).

### Study Selection

**Figure 1:**
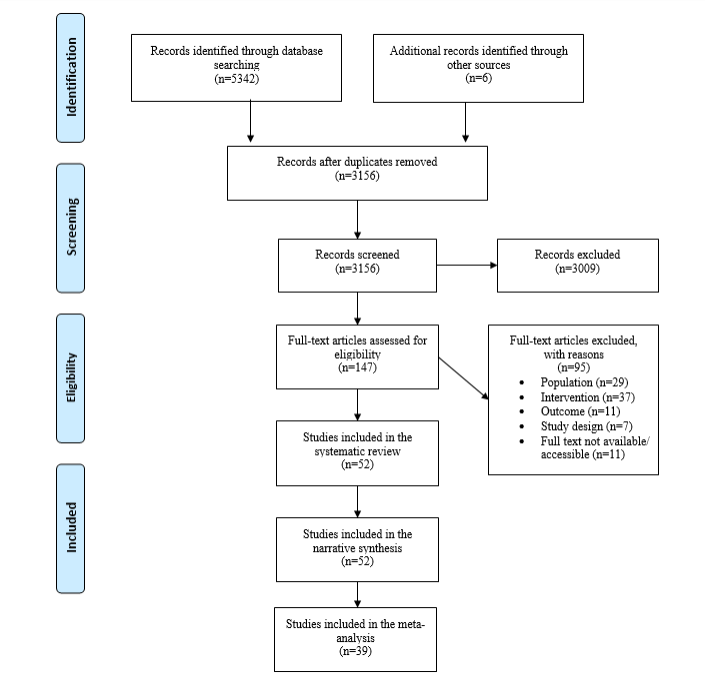
Prisma diagram of studies selected for inclusion in the systematic review and meta-analysis.

The results of the searches were downloaded and imported into the online tool CADIMA [48] for duplicates removal and study selection. All studies were independently screened by three reviewers in two stages. In the first instance, records were screened based on the inclusion/exclusion criteria, using the title and abstract. Subsequently, the full text was retrieved for the eligible articles and a full-text screening was performed. Disagreements arose for <8% of the records in both stages of the screening process. Disagreements centered around whether to include interventions that incorporated an in-person training or in-person therapy component, or what cutoff on various anxiety and stress levels was accepted for the general population. It was agreed that mild to moderate symptoms of anxiety and depression could be included but not severe symptoms. Consequently, studies including participants with scores above a clinical threshold were excluded. These disagreements were resolved through consultations with independent experts and through discussions at weekly meetings convened for the purpose of the review. Figure 1 presents the PRISMA diagram displaying the flow of records throughout the selection process.

### Quality Assessment

First, MMAT (v2018) [47] was used to assess the methodological quality of each selected study. This tool was selected based on its capacity to assess both mixed methods and quantitative studies. The tool was recently updated in 2018 and has shown evidence of good interrater reliability, usability, and content validity [47]. Studies were rated on a categorical scale as “no,” “can’t tell,” or “yes” to indicate whether they met the methodological quality criteria assessed. The number of items rated “yes” was counted to provide an overall score out of a possible 5 [47], with a higher number corresponding to stronger methodological quality. Second, the Cochrane Collaboration Risk of Bias tool (RoB-2) was applied to the identified controlled trials [49]. Each RCT was assessed for bias against six domains (ie, random sequence generation, allocation concealment, blinding of participants and personnel, blinding of outcome assessment, incomplete outcome data, and selective outcome reporting). Each domain was ranked as low risk, high risk, or unclear risk of bias. This two-fold approach was seen as a strength to present and describe the current state of the available evidence. Publication bias was assessed via visual inspection of the funnel plot asymmetry. At least two reviewers independently performed the quality assessments. The rate of agreement between the two reviewers was 56.8%. The two reviewers discussed all discrepancies and, when necessary, consulted a third team member to reach a final decision.

### Data Extraction

Data were extracted using an adaptation of the data extraction form from the Cochrane Handbook of Systematic Reviews [50]. We extracted information relating to study characteristics (eg, title, author, year of publication, aim of study, study design, type of data); participant demographic details (eg, population, setting, sample size, age, gender, ethnicity); intervention and comparator details (eg, theoretical basis, features, duration); and overall findings of the outcome measures to address the aims and objectives of this study. Two reviewers independently extracted the data and where discrepancies arose, a consensus was reached through discussions.

### Synthesis and Aggregation of Data

#### Narrative Synthesis

First, we adopted techniques from a content analytic approach [51] looking across studies to evaluate the heterogeneity of the data, similarities and differences within and between the literature and the corresponding apps, and to identify patterns or gaps within the literature. A narrative synthesis, as a textual drawing together of the findings of the studies’ and apps’ characteristics, was then performed following guidance from Popay et al [51]. Where applicable, a descriptive numerical summary is also presented to group similar articles and provide an overview of the available evidence.

#### Statistical Data Analysis

RevMan [52] was used to calculate pooled effect sizes and their 95% CIs, and to generate the corresponding figures. Guided by the Cochrane handbook [50], between-group standardized mean differences were calculated based on posttest means, SDs, and sample sizes for each of the outcomes of interest (ie, emotion regulation, well-being, and mental health). We used Hedges *g* as an index of the effect size, allowing us to include sufficiently similar outcome measures and provide adjustment for studies with small sample sizes. In agreement with the registered protocol, we included postintervention period data but excluded follow-up period data, as not all studies included a follow-up assessment, and when this was performed, it varied extensively between studies. The effect sizes were conventionally considered as small (0.2), medium (0.5), or large (0.8). Since considerable heterogeneity was expected, we used random-effects models for all analyses. Higgins *I^2^* was used as a measurement for heterogeneity [53], which was categorized as low (0-40%), moderate (30%-60%), substantial (50%-90%), or considerable (75%-100%). Sensitivity analysis was performed to test the influence of outliers and the inclusion of similar-type MHapps (eg, similar features or purpose). When necessary, we contacted primary authors for further information or used the RevMan calculator to convert the relevant data. R software using the “meta” packages were employed to estimate and account for publication bias. To estimate the risk of publication bias, funnel plots were generated and Egger statistical tests were performed for analyses with an adequate number (*k*>10) of studies [50]. When appropriate, the “trim and fill” procedures were applied to impute potential missing data and provide an adjusted estimate effect [54].

## Results

### Included Studies

A total of 52 articles [42,43,55-104] (see Multimedia Appendix 2) describing 48 interventions, published between 2008 and 2020, met the inclusion criteria. Together, the studies evaluated interventions across 15 distinct countries with a total of 22,090 research participants. Studies were mainly performed in the United States (13/52, 25%), United Kingdom (6/52, 12%), and Australia (5/52, 9.6%), with fewer studies performed in low- and middle-income countries such as Brazil (1/52, 2%) or Iran (1/52, 2%). Several studies (10/52, 19%) drew participants from a range of countries through online recruitment. Demographic profiles of the participants varied across study type and settings with the mode number of studies including participants who were younger (18-25 years; 18/52, 35%), female (44/50, 88%; gender was not reported in two studies), and of White ethnicity (18/21, 86% of the studies reporting ethnicity data). Several studies adopted an RCT study design (39/52, 75%) or a nonrandomized study design (11/52, 21%), and only a few mixed method studies were found (4/52, 8%). Study participants were mainly recruited from universities (17/52, 33%), online (15/52, 29%), or workplaces (7/52, 13%).

The rest of the Results section is organized according to the emerging evidence on mental well-being (*k*=5), mental health (*k*=10), emotion regulation (*k*=1), or any combination of these outcomes (*k*=36). This is followed by a summary of the theoretical underpinnings of the identified MHapps and an overview of the range of technological features deployed within the MHapps. Lastly, we present the results of meta-analyses that integrate the available research on the effectiveness of the identified MHapps. 

### Summary of the Emerging Research Evidence

Most of the studies adopted a randomized study design (*k*=39) and incorporated a combination or subsample of outcome measures to test effectiveness of the MHapp. Taken together, the methodological quality varied across studies (see Multimedia Appendix 2) and study samples were mainly recruited from educational settings or online. Eligibility to participate in MHapp research generally required ownership of a smart device and access to the internet. Intervention periods also varied vastly between studies (eg, 12 days versus 12 weeks). Studies captured app usage/engagement data in a range of ways, with objective app usage data only provided in less than half of the total studies (22/52, 42%). Lack of generalizability of the findings and low participant adherence to MHapp usage or the study protocol were commonly reported as limitations in the individual studies. Nonetheless, the individual studies generally reported significant positive findings for reducing mental health symptoms and/or promoting well-being or emotion regulation. A detailed overview of the emerging evidence is provided in Multimedia Appendix 3.

### Summary of the Theoretical Underpinnings of MHapps

The majority of MHapps covered in the reviewed studies were based on the theoretical principles of mindfulness, CBT, acceptance and commitment therapy (ACT), or a combination of any of these approaches, such as mindfulness-based resilience training that draws on mindfulness and ACT. In addition, positive psychology principles such as encouraging users to practice gratitude, recognize strengths, and engage in positive activities informed the design of several MHapps with the wider aim of promoting positive mental health [56]. A psychological theory specific to the treatment of particular disorders was applied in specific apps, such as providing CBT relevant to the treatment of depression or prevention of depressive thinking styles, or addressing beliefs linked with low self-esteem [42]. A detailed textual overview of the theories that are applied within the range of MHapps is provided in Multimedia Appendix 4.

### Technological Features

#### Mood Monitoring

The majority of MHapps included a mood monitoring feature where the app collects data on the user’s mood (*HeadGear, Mood Prism, MoodKit, Catch it, Pacifica, MoodHacker, Wildflowers*). Mood monitoring involved users either selecting their mood on a scale, choosing from a menu of emotions, manually inputting their mood into the app (eg, *MoodPrism*), or identifying their mood on a map (*MoodMap*) [99]. Some apps also recorded the situation where the mood was felt, the time of day, and the strength of the mood on a scale. Several MHapps also provided opportunities for users to journal or record diary entries, either responding to prompts or in free form (eg, *Oiva* and *MoodKit*) [99].

#### Assessments

Psychological assessments were built into several MHapps to enable the collection of outcome data, to inform the prescription of specific activities and exercises, and to provide data so that the user could see changes in their mood over time (*MoodPrism,* *MoodKit*) [99]. Some MHapps tracked mood and then provided an exercise or meditation (eg, *Wildflowers*). In addition, some MHapps included a risk calculator (eg, *HeadGear*) that would collect data and give users a risk score for the risk of developing a mental health condition [82].

#### Guided Meditations and Breathing Exercises

A range of studies examined MHapps that contained prerecorded mindfulness meditations, provided via an audio recording or a video clip ranging between 10 and 30 minutes, that aimed to increase the user’s capacity to practice meditation through tutorials or practice (eg, *Calm,* *Headspace, VGZ Mindfulness coach, It’s Time to Relax!, Wildflowers*). In the absence of explicit guided meditations, apps incorporated exercises to encourage users to focus their attention on their breathing alongside other features (eg, *Tactical Breather, the wellbeing mobile app*) [65].

#### Psychoeducation

Psychoeducation was delivered through in-app video, audio, and written content. Following watching or listening to psychoeducational content, users were provided with an activity, quiz, or challenge. For example, *HeadGear* set a challenge that involved planning a values-based activity or a positive activity. Many MHapps prescribed specific strategies in response to a mood that was provided via text (eg, *Jibun kiroku*) or through a suggested mindfulness exercise (eg, *Stop, Breathe, Think*). Other apps set specific social challenges to encourage users to build social connections to expand their social network or practice acts of kindness (eg, *Nod*) [64]*.*

#### Narrative Storytelling and Gamification

Some MHapps (eg, *Equoo)* used creative approaches such as storytelling. In some cases, a fictional character would convey psychoeducation or key app concepts [100]. Several apps used the metaphor of a journey and provided users with challenges or tasks (eg, *MoodMission*) [99]. One app included real-life stories of hope selected by researchers to provide examples centered around overcoming adversity [58]. Gamification was applied to promote engagement and provide rewards such as “virtual coins” for “level completion.” Gamification refers to the use of mechanisms and game-based thinking to engage users and encourage action and problem-solving [105]. In one app, gamification was used to rate the advice the user received through the app, with the user assigning points called “sprouts” (*Spring*) [101]. Several apps included quizzes to test a user’s knowledge following completing a psychoeducation module (eg, *GG Self Esteem*) [61]. Additional gamified features included earning stickers (eg, *Living with Heart*) or imaginative aspects such as placing positive messages into a virtual bottle within the app (eg, *Feel Stress Free*) [42].

#### In-App Notifications and Other Features

In-app notifications were applied in many MHapps at scheduled times and at random (eg, *Act Daily)*, which included reminders to complete psychological assessments [68]. In one app, notifications were sent containing messages of hope to promote well-being [58]. In other apps (eg, *OL@-OR@)*, tips for reducing stress and eating healthily were given via notifications [59]. Some MHapps had a virtual assistant to guide users around the app and make activity recommendations (eg, *Feel Stress Free*). Others featured a conversational agent, also described as a chatbot, to provide support to the user to adopt CBT strategies (eg, *Shim*) [56]. Although the focus of this review was on MHapps that aimed to improve emotion regulation, mental well-being, and mental health, there were some MHapps that also used sensors and trackers to detect sleep patterns (eg, *Kelaa Mental Resilience*). Other features included the analysis of the moods based on choice of music [102].

### Effectiveness of MHapps

#### Risk of Bias

**Figure 2:**
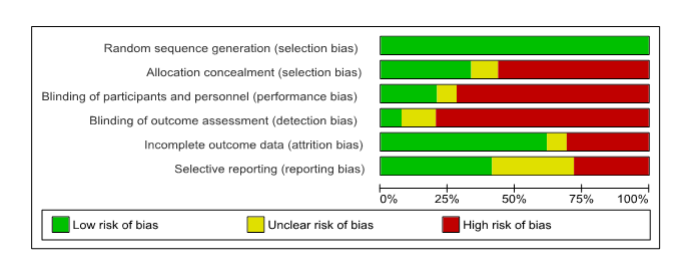
Cochrane Risk of Bias summary of randomized controlled trials included in the meta-analysis.

According to the Cochrane Risk of Bias assessment RoB-2 [49], among the 39 RCTs (including 2 mixed methods studies), 8 (21%) studies were scored as low risk in one out of six domains, 9 (23%) were scored as low risk in two out of six domains, 12 studies (31%) were rated as low risk in three out of six domains, and 5 studies (13%) were rated as low risk in four out of six domains; 4 (10%) studies were low risk in five out of six domains and 0 studies were assessed as low risk in all six domains. Studies generally were rated as high risk for allocation concealment (22/39, 56%) and blinding domains (25/39, 64%), and provided little information when reporting findings and were therefore rated unclear (19/39, 49%). All studies used an adequate randomization strategy and were thus rated as low risk. Eight studies explicitly mentioned the blinding procedures of participants and personnel. The most common method applied was random number generation via computer software; 23 studies explicitly mentioned allocation concealment. Figure 2 provides a summary of the general risk of bias of the sample of the RCTs considered for the meta-analyses. See Multimedia Appendix 5 for a summary of each study’s detailed risk of bias evaluation.

#### Well-being

**Figure 3:**
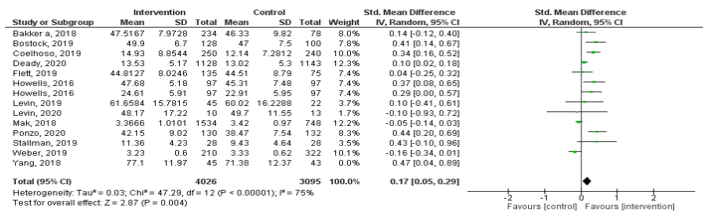
Forest plot showing the effect of MHapps vs control conditions for mental well-being.

Using data from 13 studies (Figure 3), we compared the effects of MHapps with any control group (eg, assessment only, waitlist, treatment as usual, or active control groups). The meta-analysis revealed a very small pooled effect size (*g*=0.17, 95% CI 0.05-0.29, *P=*.004) in favor of MHapps having the potential to improve well-being. However, a considerable amount of heterogeneity was present (*I^2^*=75%). Five studies were excluded based on our planned sensitivity analysis. Two studies were judged as outliers [71,88]. Three studies were judged to be dissimilar in purpose and/or features of the MHapp or outcome measures, or had insufficient data available [58,74,89]. The Egger test was not significant and therefore trim-and-fill procedures were not applied [44].

#### Mental Health

**Figure 4:**
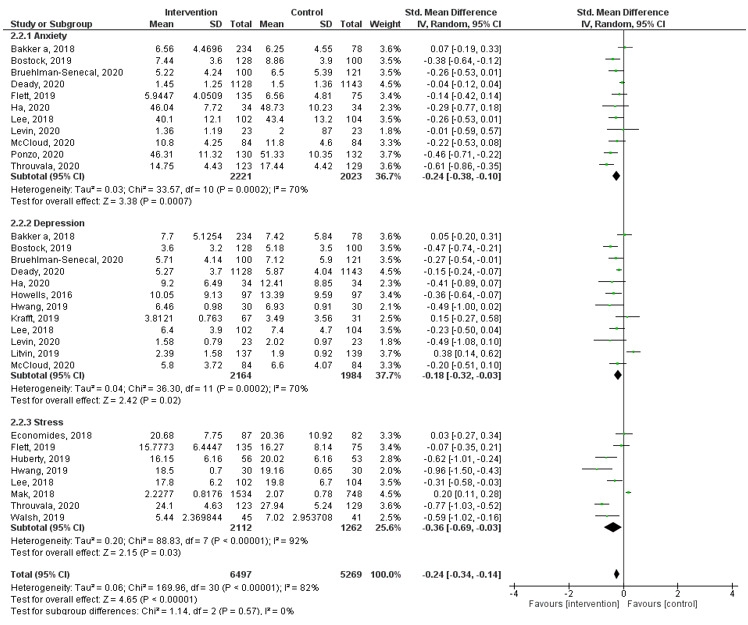
Forest plot of effect of MHapps versus a control condition on anxiety, depression, and stress.

Using data from 19 studies (Figure 4) revealed an overall small pooled effect (*g*=–0.24, 95% CI –0.34 to –0.14, *I^2^*=82%, *P*<.001) with a considerable amount of heterogeneity, indicating the potential of MHapps to lower any of the mental health symptoms (ie, anxiety, depression, stress) when compared with controls. Subgroup analyses indicated that MHapps had a small pooled effect on reducing stress (*g*=–0.36, 95% CI –0.69 to –0.03, *I^2^*=92%). For reducing anxiety symptoms, MHapps had a small pooled effect (*g*=–0.24, 95% CI –0.38 to –0.10, *I^2^*=70%), and for reducing depression symptoms, MHapps had a very small pooled effect (*g*=–0.18, 95% CI –0.32 to 0.03, *I^2^*=70%). However, substantial or considerable heterogeneity was observed among the studies. Eight studies were excluded based on our planned sensitivity analysis. Four studies were judged as outliers [72,80,81,88]. Four studies were judged to be dissimilar in purpose and/or features of the MHapp or outcome measures used, or had insufficient data available [67,71,74,98]. An examination of the funnel plot and a significant Egger test indicated a high level of potential publication bias. After trim-and-fill procedures were applied, adding 17 potential studies (see Appendix 6, Figure S3), the overall pooled effect of MHapps for reducing mental health symptoms was no longer significant (*g*=0.08, 95% CI –0.17 to 0.34, *P*=.50).

#### Emotion Regulation

**Figure 5:**
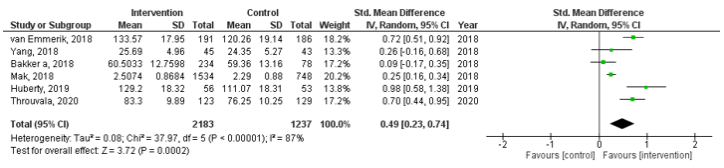
Forest plot showing the effect of MHapps versus a control condition on the outcome of emotion regulation.

Using data from 6 studies (Figure 5) revealed a medium effect (*g*=0.49, 95% CI 0.23-0.74) in favor of MHapps having the potential to improve emotion regulation over control conditions. A considerable amount of heterogeneity was also present (*I^2^*=87%, *P<*.001). This analysis included less than 10 studies (*k*=6) and therefore the Egger test was deemed inappropriate [50]. Owing to methodological weaknesses and unreliability of their estimates, trim and fill was also not attempted [44].

#### Other Important Findings

None of the reviewed studies reported well-being and mental health apps to be harmful or to have any kind of adverse effect on users. Similarly, we observed that none of the reviewed studies reported on cost-effectiveness.

## Discussion

### Summary of Findings

This review documents the emerging evidence on available MHapps that promote emotion regulation, well-being, and mental health in the general population. Fifty-two articles describing and evaluating 48 MHapps met the inclusion criteria, with a total of 39 RCTs identified. Overall, the review found that there is growing evidence and support for the role of MHapps in improving and promoting positive mental health, emotion regulation, and well-being in the general population. Results from the meta-analyses of RCTs indicated significant small effects of MHapps compared to control conditions for well-being, symptoms of stress, depression, and anxiety. The meta-analysis also indicated a significant medium effect of MHapps compared to control conditions for emotion regulation. There is also a foundation of theoretically based empirical studies documenting creative and innovative ways to engage users; mindfulness and cognitive behavioral approaches appear to be the most common among app developers.

Based on the reviewed studies, the evidence on mobile apps to promote emotion regulation, well-being, and mental health in the general population is still in its infancy. A fair proportion of studies were pilot or feasibility trials (17/52, 33%), and full-scale RCTs reported high attrition rates and nondiverse samples, limiting the extent to which the findings could be generalized. The evidence is also limited, with few studies on MHapps specifically for emotion regulation in the target population, and on safety and cost-effectiveness related to mobile apps, which are important components of digital interventions. Moreover, heterogeneity was generally substantial and large confidence intervals were observed in many studies. In the same vein, the lack of qualitative data limits our understanding of in-depth user experience. Lastly, techniques and methods used in MHapp research varied vastly in terms of intervention period, adherence measurements, and recruitment strategies, with implications for pooling the findings to assess overall effectiveness.

### Comparison to Other Reviews

This study adds to the review performed by Wang et al [16] suggesting that MHapps have potential to be effective in monitoring or improving symptoms of certain mental disorders. The findings also extend recent findings by Gál et al [35] that suggested the potential for mindfulness MHapps to improve well-being. Specifically, our findings add that the potential may extend beyond mindfulness-focused MHapps, while also highlighting the potential for success within a specific target audience (18-45 years old). Our findings also align with previous evidence suggesting that apps are important for mood management, improving mental well-being, and life satisfaction through better management of emotions [14]. In the absence of imputed data, the current findings also suggested small to medium pooled effect sizes for mental health, including anxiety, depression [17], and stress [17,106]. Similarly, small pooled effects were repeated for well-being [35]. Among MHapp research, heterogeneity appears to be consistently high [39], as was repeated in this study.

As per the systematic review of distant mood monitoring apps by van der Watt et al [107], participants included in the present review were mostly female. In the previous review, the authors suggested that this gender bias is related to the higher rates of depressive and bipolar disorders in women [107]. However, an in-depth understanding of why samples tend to have a majority of female participants would be important future steps to meet the needs of men and other genders who may be currently underserved [107]. In accordance with other reviews, our findings also highlighted that none of the identified MHapps was harmful to users [108].

### Interpretation of the Findings

Given that the extant literature shows that the effectiveness of the vast majority of available MHapps is not well supported by evidence-based research [33], this review has provided evidence of a range of mental well-being, mental health, and emotion regulation apps that are supported by the literature. There is substantial empirical support for MHapps that apply theoretical insights from CBT and mindfulness approaches. Mood monitoring in various forms is also well-supported empirically, as was ecological momentary assessment and the value of users recording moods as part of their daily lives. Other approaches are much less supported from a small number of empirical studies and warrant further research.

This review found a lack of empirical studies investigating the effectiveness of apps for emotion regulation for adults. Although there is substantial literature addressing the impact of virtual reality emotion regulation interventions for well-being and emotion regulation in adults, and video games in children [109,110], the effectiveness of MHapps that aim specifically to support emotion regulation in adults has not been systematically investigated. Notwithstanding the substantial amount of heterogeneity, this review found a medium effect of MHapps on emotion regulation, suggesting a promising area for further exploration.

Interestingly, within the reviewed MHapps, some strategies employed for well-being and mental health outcomes are also relevant to emotion regulation. For example, cognitive reappraisal is a common emotion regulation strategy [111]. Cognitive reappraisal was taught via psychoeducation in several apps, but emotion regulation was rarely a primary outcome in the studies or among the aims of the included MHapps. Similarly, mood monitoring has been found to increase emotional self-awareness, and there is some evidence to suggest that a low level of self-awareness is a risk factor for anxiety, depression, and stress [26,112]. Thus, increasing emotion regulation has been explored as an approach to improve mental health and reduce the risk of mental health disorders [112].

From the current evidence, beyond mood monitoring and in-app meditations, it is difficult to say which specific features are the most effective in MHapps. Additional features such as gamification, use of virtual assistants, rewards, and chatbots were also observed in some of the studies, albeit these were assessed in a relatively less systematic manner. Moreover, some apps adopted innovative approaches such as “crowdsourcing” therapeutic advice, in-app rating of the advice received, and use of music to assist users with moods. These less-studied approaches would also benefit from further research. In particular, the use of mixed methods designs could prove particularly helpful for illustrating the limitations of a particular feature or the limitations of technology in general as a source of provision of psychological support.

### Implications for Research

It is clear that app content, design, and features, including the underpinning theory of change and the mode of delivery, will influence engagement with the app and, by extension, its impact on an individual. We agree with Hollis et al [113] that this can make it difficult to judge whether outcomes of a study are associated with the intervention content and theory of the change, the digital delivery platform, or an interaction between the two. It may be recommended that implementation science account for these dynamic and sometimes complex designs, and include components that are capable of evaluating individual differences. Equally, it is important that studies seek to understand the mechanisms such as particular features or theories that contribute to changes in user outcomes in further research.

Adherence and retention continue to be challenges to the quality of research, with little or no information about reasons for dropouts given across studies. Research designs may also need to adapt to capture such information that might be needed to inform future trials of similar apps. For example, follow-ups on dropouts and inclusion of insights from qualitative data might provide vital information. Although some studies reported objective app use data (eg, [68]), other studies relied on user reports of app usage. It is anticipated that app research would benefit from reporting more objective app use data. Another important point to consider is the high heterogeneity in the measure of retention/adherence across different MHapps. In the absence of standardized measures, researchers should aim to justify the choice of specific adherence/retention measures and how they can best be adapted to the app designs. For example, adhering to an intervention for a certain period (eg, 2 weeks or 1 month) may be different from completing a set number of modules or tasks. Similarly, usage of 10 minutes per day compared to “logging on” at least once during the intervention period could impact how results are interpreted. These variations may further implicate how findings can or should be pooled together to inform future reviews to assess overall effectiveness.

Alongside a general need for more RCTs and qualitative or mixed methods studies, this review identified a dearth of evidence on apps aiming to improve emotion regulation (despite mood management being integrated within many apps). Another finding of this review was an absence of diverse samples in included studies; thus, researchers should aim to address the lack of variance in ethnicity and socioeconomic status of the populations included in the current literature. In particular, studies should focus on minority populations, and should be performed in both low- and middle-income countries to aid with generalizability and to identify any differences in MHapp effectiveness or engagement for users from different demographic backgrounds.

### Implications for App Development

The increased use of digital devices to support mental health suggests that MHapps are likely to become a relevant aspect of a proactive mental health and well-being model in the next few years. There is substantial positive support for the model of an app that captures the user’s moods or emotional states and then provides information, and there is support for apps that provide a short, regular meditation session (eg, *Calm*). It is recommended that developers continue to implement these strategies with a focus on engagement. We agree with Huberty et al [114] that it is helpful to take the different stages of user engagement into account as part of app development (eg, mood check-ins). Nonetheless, some features continue to be underexplored and underused (eg, chatbots, diaries, games, storytelling, rewards, crowdsourcing, avatars, personalization); such features may have the potential to be effective in other digital interventions. In particular, there is potential for the development of features (eg, notifications and gamification) that can improve retention in both app use and studies.

Moreover, despite the proliferation of MHapps, a wider range of psychological theories (eg, attachment theory) could be explored and incorporated to broaden our understanding of applying technology to achieve improved outcomes and therapeutic change via MHapps. It may be just as important to include researchers and other relevant stakeholders in the early stages of the design process to ensure that the prototypes are useful and usable for a wider audience and will be recommended or endorsed by experts. Equally, it is also important to continue to include end users as part of the human-computer interaction approach to app development.

### Strengths and Limitations

This review followed established guidelines for performing systematic reviews [50]. The protocol for the review was published on PROSPERO and search terms were reviewed with an experienced university librarian at University College London. As recommended, the screening, quality assessment, and data extraction processes were undertaken by at least two independent reviewers and checked by a senior researcher to reduce the chance of selection bias or omitting any relevant information. Although several measures were in place to ensure that a rigorous systematic review process was undertaken, this review is not devoid of limitations. This study may have been limited by the search terms used. There is a wide range of terminology used to describe apps; multiple definitions of well-being, mental health, and emotion regulation; and many well-being mediators. There were also challenges in separating standalone apps versus human-mediated interventions, as this was not always clearly stated or we were not aware of the extent to which phone calls, emails, and text messages were related to the study design. Similarly, although we excluded studies and MHapps targeting formally diagnosed mental health clients, an objective definition of “general population” is not straightforward. As a result, we could have unknowingly failed to identify and include relevant articles.

Lastly, studies were judged to be of varying quality across the different categories and of high risk of bias. Based on the findings of this review, several other sources of bias may also need to be considered in future studies. For example, the differences in sample demographics should be incorporated, which may not always be discernible from published results, but may contribute to differences in response rates. As a result, our findings should be interpreted in light of these shortcomings.

### Conclusions

This systematic review focused on the relatively less studied but important domain of MHapps for mental well-being, positive mental health, and emotion regulation. The review found 52 publications describing 48 MHapps. When pooled, the MHapps demonstrated a small effect for reducing mental health symptoms and improving well-being and a medium effect for increasing emotion regulation. The findings of the meta-analysis suggest that MHapps have potential to assist users to manage mental health symptoms, boost well-being, and foster emotion regulation. Therefore, MHapps may be an important source of mental health care in the current climate of increased rates of mental health disorders and poor well-being.

In terms of the current state of the evidence for MHapps, existing research indicates some benefits to app usage, and several creative and innovative ways to engage and reward users over time. Such features enable users to learn and apply psychoeducational content, learn mindfulness or other techniques, as well as to input changes in moods and emotions. However, there remain some areas for further development within the evidence base. The body of knowledge could benefit from more large-scale RCTs and qualitative research with diverse research samples, consistent and standardized approaches to measurements (eg, reporting objective app use data), further granularity on which features are effective and how, and a specific focus on apps that support users with emotion regulation. More evidence-based apps incorporating multiple psychological theories and other innovative modes of delivery are also welcomed. Researchers, app developers, end users, and other relevant stakeholders should continue to work together to ensure that the apps are not only effective but also useful, usable, safe, cost-efficient, and sustainable over time.

## Appendix

Multimedia Appendix 1 Search strategy.

Multimedia Appendix 2 Characteristics of included studies.

Multimedia Appendix 3 An overview of the emerging evidence for MHapps.

Multimedia Appendix 4 An overview of the theoretical underpinning of the reviewed MHapps.

Multimedia Appendix 5 Risk of bias summary.

Multimedia Appendix 6 Funnel plots for mental health, mental well-being and emotion regulation outcomes.

## Abbreviations

ACT: acceptance and commitment therapy
CBT: cognitive behavior therapy
MHapp: mental health app
MMAT: Mixed Methods Appraisal Tool
PRISMA: Preferred Items for Reporting a Systematic Review and Meta-analysis
PTSD: posttraumatic stress disorder
RCT: randomized controlled trial
RoB: Risk of Bias
