# Supplementary material for: Mobile Apps That Promote Emotion Regulation, Positive Mental Health, and Well-being in the General Population: Systematic Review and Meta-analysis
Source: JMIR Ment Health. 2021 Nov 8;8(11):e31170. doi: 10.2196/31170 (PMC8663676; doi:10.2196/31170)
Supplement: Multimedia Appendix 1 [file mental_v8i11e31170_app1.docx]

**Appendix 1**

Systematic Review Search strategy (TI/AB/KW)

Searches

Terms are searched on abstracts, titles, keywords, and subheadings and are organized around:

- INTERVENTION (digital intervention terms) and
- multiple OUTCOME themes (Wellbeing OR Emotion) and
- Type of study (RCTs or before-after)

MED WoS EMB Coc Psy

**Topic: outcome (wellbeing and emotion regulation) - search on AB/TI/KW**

1. "mental health"; mentalhealth MED WoS EMB Coc Psy
2. wellbeing; "well being"; well-being MED WoS   EMB Coc Psy
3. "positiv* affect" MED WoS EMB Coc Psy
4. "life satisfaction" MED WoS EMB Coc Psy
5. happ* MED WoS EMB Coc Psy
6. "coping behaviour"; "coping behavior" MED WoS EMB Coc Psy
7. "work-life balance";"work life balance" MED WoS EMB Coc Psy
8. resilience MED WoS EMB Coc Psy
9. "quality of life"  MED WoS EMB Coc Psy
10. HRQOL MED WoS EMB Coc Psy
11. "Emotion* aware*" MED WoS EMB Coc Psy
12. "Emotion* fit*" MED WoS EMB Coc Psy
13. "Emotion* intelligen*" MED WoS EMB Coc Psy
14. "emotion* regulation" MED WoS EMB Coc Psy
15. "emotion* control" MED WoS EMB Coc Psy
16. "emotion* management" MED WoS EMB Coc Psy
17. "emotion* stab*" MED WoS EMB Coc Psy
18. "emotion* security" MED WoS EMB Coc Psy
19. “emotion* adjustment” MED WoS EMB Coc Psy
20. “emotion* function” MED WoS EMB Coc Psy
21. “Selfcare”; “self care”; self-care MED WoS EMB Coc Psy
22. “Wellness”; “well ness”;well-ness MED WoS EMB Coc Psy

AND

**Topic: intervention (mobile app) - search on AB/TI/KW**

1. "smartphone technology" MED WoS EMB Coc Psy
2. "smartphone app*" MED WoS EMB Coc Psy
3. mobile app* MED WoS EMB Coc Psy
4. "mobile phone app*" MED WoS EMB Coc Psy
5. "mobile device app*"  MED WoS EMB Coc Psy
6. "portable software app*" MED WoS EMB Coc Psy
7. "tablet app*" MED WoS EMB Coc Psy
8. "digital intervention" MED WoS EMB Coc Psy
9. "digital treatment" MED WoS EMB Coc Psy
10. "digital app*" MED WoS EMB Coc Psy
11. "Mapp" or “M app” MED WoS EMB Coc Psy
12. MHapp or "MH app" MED WoS EMB Coc Psy
13. DMHI MED WoS EMB Coc Psy
14. "digital mental health intervention"  MED WoS EMB Coc Psy
15. Telepsychology MED WoS EMB Coc Psy
16. Mhealth MED WoS EMB Coc Psy

Removed:

1. Digital (this has to be removed: fingers) WoS Coc

 AND

**Topic: type of study - search on all fields**

1. RCTs or RCT   MED WoS EMB Coc Psy
2. "randomi?ed control* trial*"  MED WoS EMB Coc Psy
3. "randomi?ed control* stud*" MED WoS EMB Coc Psy
4. beforeafter/before after/before-and-after stud* MED WoS EMB Coc Psy
5. beforeafter/before after/before-and-after design MED WoS EMB Coc Psy
6. pretest/pre test posttest/post test stud* MED EMB Psy
7. pretest/pre test posttest/post test design MED EMB Psy
8. Quasi experiment*; quasi-experiment* MED WoS EMB Coc Psy
9. “cross over trial”; cross-over trial MED WoS EMB Coc Psy
10. Control* trial* MED WoS EMB Coc Psy
11. Randomi?ed MED WoS EMB Coc Psy
12. pre$test MED WoS EMB Coc Psy
13. Post?test MED WoS EMB Coc Psy
14. Clinic* trial* MED WoS EMB Coc Psy

**Web of Science= 1025 (05/11/2020)** *removed digital - English only -2008-2020*

**TOPIC**: ("mental$health" or "mental health" or "self$care" or "self care" or "emotion* adjustment" or "well$ness" or "well ness" or well$being or "well being" or "emotion* regulation" or "emotion* function" or "emotion* control" or happ* or "positiv* affect" or "life satisfaction" or "coping behavio$r" or "work life balance" or "work-life balance" or resilience or "quality of life" or HRQOL or "emotion* aware*" or "emotion* fit*" or "emotion* intelligen*" or "emotion* control" or "emotion* management" or "emotion* stab*" or "emotion* security")

**TOPIC**: "smart$phone technolog*" or "smart$phone app*" or “mobile app*” or "mobile phone app*" or "mobile device app*" or "portable software app*" or "tablet app*" or “digital intervention” or "digital treatment" or "digital app*" or "M$app" or "M app" or "MH$app" or "MH app" or  "digital mental health intervention" or DHMI or  tele$psychology or m$health

**ALL FIELDS**: "randomi?ed control* trial" or RCT* or "randomi?ed control* stud*" or "beforeafter study" or "beforeafter design" or "before and after study" or "before and after design" or "pre*post*" or pre$test or “pre test” or post$test or “post test” or "quasi$experiment*" or "quasi experiment*" or randomi?ed or "cross$over trial*" or “control* trial*” or “clinic* trial*”

**Cochrane Library= 1560 (05/11/2020) trials** *-2008-2020*

**Title Abstract Keyword**: "mental*health"or "self*care" or "emotion* adjustment" or "well*ness" or well*being or "emotion* regulation" or "emotion* function" or "emotion* control" or happ* or "positiv* affect" or "life satisfaction" or "coping behaviour" or "coping behavior"  or "work life balance" or resilience or "quality of life" or HRQOL or "emotion* aware*" or "emotion* fit*" or "emotion* intelligen*" or "emotion* control" or "emotion* management" or "emotion* stab*" or "emotion* security"

**Title Abstract Keyword**: "smart*phone technolog*" or "smart*phone app*" or  mobile app* or "mobile phone app*" or "mobile device app*" or "portable software app*" or "tablet app*" or "digital treatment" or “digital intervention” or  "digital app*" or "M*app" or "MH*app" or "digital mental health intervention" or DHMI or tele*psychology or m*health

**ALL Text**: "randomi?ed control* trial" or RCT* or "randomi?ed control* stud*" or "before and after study" or "before and after design" or "pre*post*" pre*test or post*test or "quasi*experiment*" or randomi?ed or "cross*over trial*" or “control* trial*” or “clinic* trial*”

**Mesh Terms search**

((mental health or happiness or work-life balance or adaptation, psychological or resilience, psychological or quality of life or emotional adjustment or emotional regulation or emotional intelligence) and (mobile applications) and (randomized controlled trials as topic or non-randomized controlled trials or controlled before-after studies or controlled clinical trials as topic)).mh.

**Ovid(MEDLINE)= 902 (05/11/2020)** [672 results without words in red] *- 2008-2020*

**Title Abstract Keyword**: (mentalhealth or mental health or selfcare or self care or wellbeing or well being or wellness or well ness or positiv* affect or life satisfaction or happ* or coping behavio*r or work-life balance or resilience or quality of life or HRQOL or Emotion* aware* or Emotion* fit* or Emotion* intelligen* or emotion* regulation or emotion* control or emotion* management or emotion* stab* or emotion* security or emotion* function)

**Title Abstract Keyword**: (smartphone technolog* or smart phone technolog* or smartphone app* or smart phone app* or mobile app* or mobile phone app* or mobile device app* or portable software app* or tablet app* or digital intervention or digital treatment or digital app* or M app or Mapp or MHapp MH app or DMHI or digital mental health intervention or telepsychology or mhealth)

**All fields**: (RCTs or RCT or randomi?ed control* trial or randomi?ed control* stud* or beforeafter stud* or before after stud* or beforeafter design or before after design or before-and-after study or before-and-after design or pretest posttest study or pre test post test study or pretest posttest design or pre test post test design or time-series stud* or quasi experiment* or cross over trial* or control* trial or randomi?ed or Clinic* trial* or pretest or pre test or posttest or post test)

OR

**Mesh Terms search**

((mental health or happiness or work-life balance or psychological resilience or quality of life or emotional regulation or emotional intelligence) and (Mobile applications) and (randomized controlled trials as topic or non-randomized controlled trials as topic or cross-over studies or clinical trials as topic))

**Ovid(EMBASE)= 1188 (05/11/2020)** [887 results without words in red] *- 2008-2020*

**Title Abstract Keyword**: (mental health or mentalhealth or wellbeing or well being or positive affect or life satisfaction or happ* or coping behavio*r or work-life balance or resilience or quality of life or HRQOL or Emotion* aware* or Emotion* fit* or Emotion* intelligen* or emotion* regulation or emotion* control or emotion* management or emotion* stab* or emotion* security or emotion* adjustment or emotion* function or self care or selfcare or wellness or well ness)

**Title Abstract Keyword**: (smartphone technology or smart phone technology or smartphone app* or smart phone app or mobile app* or mobile phone app* or mobile device app* or portable software app* or tablet app* or digital intervention or digital treatment or digital app* or Mapp or M app or MH app or MHapp or DMHI or digital mental health intervention or telepsychology or mhealth)

**All fields**: (RCTs or RCT or randomi?ed control* trial* or randomi?ed control* stud* or beforeafter stud* or before after stud* or beforeafter design or before after design or before-and-after study or before-and-after design or pretest posttest study or pre test post test study or pretest posttest design or pre test post test design or time-series stud* or quasi experiment or cross over trial or control trial or randomi?ed or Clinic* trial* or pretest or pre test or posttest or post test).af.

OR

**Mesh Terms search**

((mental health or wellbeing or psychological well-being or life satisfaction or happiness or coping behavior or work-life balance or psychological resilience or quality of life or emotion regulation or emotional intelligence) and (mobile application) and (randomized controlled trial or pretest posttest control group design or controlled study))

**Ovid(APA PsycInfo)= 539 (05/11/2020)** *- 2008-2020*

**Title Abstract Keyconcepts**: (mental health or mentalhealth or wellbeing or well being or positive affect or life satisfaction or happ* or coping behavio*r or work-life balance or resilience or quality of life or HRQOL or Emotion* aware* or Emotion* fit* or Emotion* intelligen* or emotion* regulation or emotion* control or emotion* management or emotion* stab* or emotion* security or emotion* adjustment or emotion* function or self care or selfcare or wellness or well ness).ab,ti,id.

**Title Abstract Keyconcepts**: (smartphone technology or smart phone technology or smartphone app* or smart phone app or mobile app* or mobile phone app* or mobile device app* or portable software app* or tablet app* or digital intervention or digital treatment or digital app* or Mapp or M app or MH app or MHapp or DMHI or digital mental health intervention or telepsychology or mhealth).ab,ti,id.

**All fields**: (RCTs or RCT or randomi?ed control* trial* or randomi?ed control* stud* or beforeafter stud* or before after stud* or beforeafter design or before after design or before-and-after study or before-and-after design or pretest posttest study or pre test post test study or pretest posttest design or pre test post test design or time-series stud* or quasi experiment or cross over trial or control trial or randomi?ed or Clinic* trial* or pretest or pre test or posttest or post test).af.

OR

**Mesh Terms search**

((mental health or happiness or work life balance or resilience psychological or quality of life or emotional adjustment or emotional regulation or emotional intelligence) and (mobile applications) and (randomized controlled trials as topic or non randomized controlled trials as topic or controlled before after studies or controlled clinical trials as topic)).mh.
