# Supplementary material for: Mobile Apps That Promote Emotion Regulation, Positive Mental Health, and Well-being in the General Population: Systematic Review and Meta-analysis
Source: JMIR Ment Health. 2021 Nov 8;8(11):e31170. doi: 10.2196/31170 (PMC8663676; doi:10.2196/31170)
Supplement: Multimedia Appendix 2 [file mental_v8i11e31170_app2.docx]

Appendix 2

Characteristics of included studies

| **Author and year of study** | **Intervention** | **Theoretical underpinning** | **Study design** | **Study aims** | **Country** | **Recruitment setting** | **Sample size** | **Demographic information** | **Reported Findings of App Effectiveness** | **QA score** |
| --- | --- | --- | --- | --- | --- | --- | --- | --- | --- | --- |
| Ahtinen et al. (2013) | *Oiva*  A wellbeing app that uses ACT to help users manage stress. App includes psychoeducation via video and text, with exercises to complete and a diary to aid reflection. | ACT | Mixed methods | To study the usage, acceptance, and usefulness of a wellbeing app among working-age individuals | Finland | University/College | 15 | 5 participants were younger than 30 years old, 5 were between 31 and 40 years, and 5 were older than 40 years, 60% female. | Significant pre-post improvements were obtained in stress ratings and satisfaction with life scores, but not in psychological flexibility. | 5 |
| Arean et al. (2016) | *Project: EVO*  *A* video game app that encourages cognitive control. *iPST* is a problem-solving therapy app that aims to lower depression. | Combination | RCT | To compare use patterns and clinical outcomes between 2 depression apps and a control. | USA | Online | 626 | 33.9 (11.84); 79% female; 410 White (65.5%) | Differential treatment effects were present in participants with baseline PHQ-9 score >10, with the cognitive training and problem-solving apps resulting in greater effects on mood than the information control app. | 2 |
| Bakker & Rickard (2018) | *MoodPrism*  App tracks users' moods over time and collects information about users' emotional states (via assessments). | CBT | Nonrandomised | To evaluate the effectiveness of MoodPrism and study the mediators of mental health and wellbeing outcomes. | Global | Online | 234 | 34.8 (14.2), range 13-69, 72% female, 65% completed tertiary education | In this community sample, app engagement ratings predicted decreases in depression and anxiety, and increases in mental well-being. These effects were mediated by increases in emotional self-awareness, but only for participants who were clinically depressed or anxious at the time of the baseline assessment. Engaging with an emotional wellbeing self-monitoring app may reduce depressive and anxious symptoms, and increase mental well-being. | 2 |
| Bakker et al. (2018) | *MoodKit, MoodPrism & MoodMission* MoodPrism tracks users' moods over time and collects information about users' emotional states (via assessments).  MoodMission: Users input their current emotional distress and app provides a list of five CBT-based activities, called “Missions”, for users to choose. MoodKit is a CBT-based app that contains four main tools: a collection of activities, a thought checker, a mood tracker, and a journal." current emotional distress and MoodMission app provides a list of five CBT-based activities, called “Missions”, for users to choose. | CBT | RCT | To evaluate the superiority of the three MHapps to a waitlist control condition | Australia | Online | 312 | 34.2 (12.1), range 18-76, 81% female | Compared to the control condition, all MHapp groups experienced increases in mental wellbeing, MoodKit and MoodMission groups experienced decreases in depression, and no groups experienced effects on anxiety. Mediated regressions revealed that increasing coping self-efficacy, rather than emotional self-awareness or mental health literacy, was the underlying process contributing to effects on mental health for all three MHapps. | 1 |
| Bakker, D., & Rickard, N. (2019) | *MoodMission* Users input their current emotional distress and *MoodMission* app provides a list of five CBT-based activities, called “Missions”, for users to choose. | CBT | Nonrandomised | To investigate the relationships between mental health outcomes and app engagement. | Global | Online | 617 | 26.9 (10.9), range 13-70, 71% female, 65% completed tertiary education, 34% employed part-time | App engagement ratings predicted increases in mental wellbeing. Mediation analyses revealed that there were indirect effects of app engagement on depression, anxiety, and mental wellbeing via the mediator of coping self-efficacy.  Subsample analyses found this only for participants who were experiencing a moderate level of depression or anxiety at the time of the baseline assessment. | 3 |
| Birney et al. (2016) | *MoodHacker* App that encourages positive activity planning and tracking, cognitive restructuring, mindful self-awareness, gratitude expression, and identifying and utilizing strengths). | Other | RCT | To demonstrate the efficacy of MoodHacker with working adults with mild-to-moderate depression. | USA | Online | 300 | 40.65 (11.35), 85% Non-Hispanic/Latino, 76.65% female, 58.65% employed full time, 50% married/living with a partner | At 6-week follow-up, significant effects were found on depression, behavioral activation, negative thoughts, knowledge, work productivity, work absence, and workplace distress. MoodHacker yielded significant effects on depression symptoms, work productivity, work absence, and workplace distress for those who reported access to an EAP, but no significant effects on these outcome measures for those without EAP access. Significant effects on work absence in those with EAP access persisted at 10-week follow-up. | 4 |
| Borjalilu et al. (2019) | *Aramgar*  The stress management application was designed based on emotion-focused coping according to the mindfulness-based stress reduction (MSBR) | Mindfulness | Nonrandomised | To compare the effectiveness of three interventions for stress management in students. | Iran | University/College | 68 | 24.29 (3.21), 71% female, 47% single, 29% employed | There was a significant difference in the mean score reduction of depression, stress, and anxiety between the three groups. The post hoc test showed that the blended therapy group had the greatest mean score reduction on stress, depression, and anxiety among the three groups. | 1 |
| Bostock et al. (2019) | *Headspace* In app mindfulness training program consisting of 45 meditation sessions lasting from 10 to 20 min. | Mindfulness | RCT | To examine the effects of Headspace on work stress and other outcomes. | UK | Workplace | 238 | 35.5 (7.7), range 23-61, 59.2% female, 96% employed full time. | The intervention group reported significant improvement in well-being, distress, job strain, and perceptions of workplace social support compared to the control group. In addition, the intervention group had a marginally significant decrease in self-measured workday systolic blood pressure from pre- to postintervention. Sustained positive effects in the intervention group were found for well-being and job strain at the 16-week follow-up assessment. | 3 |
| Bruehlman-Senecal et al. (2020) | *Nod* app that includes social challenges, reflections and written testimonials to increase social connections. | Combination | RCT | To examine the initial efficacy, feasibility, and desirability of Nod app, designed to reduce loneliness. | USA | University/College | 221 | 18.68 (0.35), range 18-20, 59.3% female, 52.9% White, 42.3% middle class, 66.1% heterosexual, 78.7% single | There were no significant condition differences in loneliness at week 4. However, there was a significant condition-by-baseline depression interaction to predict week-4 loneliness. Simple slope analyses indicated that baseline depression positively predicted week-4 loneliness among control participants, but not among experimental participants, suggesting that Nod buffered participants with high baseline depression scores from experiencing heightened midquarter loneliness. Similarly, there were no significant condition differences in other week-4 outcomes. However, moderation by baseline vulnerability was found for week-4 depressive symptoms, sleep quality, and indices of college adjustment. | 3 |
| Carissoli et al. (2015) | *It's Time to Relax!* In this app participants practice two mindfulness meditations per day, lasting 15 minutes each, by listening to the guided or free meditation. | Mindfulness | RCT | To examine the efficacy of a mindfulness inspired protocol,  in reducing stress in adults. | Italy | Workplace | 56 | M = 38.11, SD = 6.92, range 20-52, 79% women, 66.1% had a University degree, 66.1% were white-collar workers | The results did not show any significant differences between groups, but both self-help intervention groups  demonstrated an improvement in coping with stress. | 2 |
| Champion et al. (2018) | *Headspace* In app mindfulness training program consisting of 45 meditation sessions lasting from 10 to 20 min. | Mindfulness | RCT | To replicate and extend a previous study (Howells et al., 2014). | UK | Global | 74 | 34.9 (5.76), range 25-59, 55% female, 97% based in the UK | Relative to the WL control, the MM app positively impacted self-reported satisfaction with life, stress, and resilience at day 10, with further improvements emerging at day 30. The rate of improvement was largest at the 10-day assessment point, dropping moderately by day 30. Moreover, the MM app was able  to protect against an unexpected increase in perceived stress that emerged in the control group. | 3 |
| Coelhoso et al. (2019) | *The wellbeing mobile app* 8 week in app program based on relaxation training, breathing techniques, guided meditation, and positive psychology principles. | Mindfulness | RCT | To evaluate the effectiveness of a wellbeing app among working women. | Brazil | Health Centre or Health Service | 490 | 34.6 (7.62), 100% female, 53% married/lived with a domestic partner, 45% had a graduate degree | Both groups showed a significant increase in general well-being as a function of time, but only the intervention group presented a significant increase in work-related well-being, as well as a significant reduction in work-related and overall stress. | 4 |
| Daugherty et al. (2018) | *Real Life Exp*  App delivering hope notifications at random to promote wellbeing (hope picture, hope statement and question) and capturing mood information via EMI. | EMI | RCT | To examine whether a hope ecological momentary intervention increased hope and wellbeing. | USA | University/College | 112 | range 18-25, 70.5% female, 88.4% White Non-Hispanic | Relative to the control group, those receiving the intervention demonstrated significantly greater increases in hope; however, there were no between-group differences in hedonic and eudaimonic well-being. | 1 |
| Deady et al. (2018) | *HeadGear*  App that offers a risk assessment for mental health issues followed by a daily challenge for 30 days on topics such as coping skills, mindfulness provided in app. | Mindfulness | Nonrandomised | To evaluate the usability and preliminary efficacy of the HeadGear app in a male dominated industry working population. | Australia | Workplace | 84 | 38.0 (9.23), 100% men, 54% working in nonmale dominated industry, 67% general employees | Stage 2 found that compared with baseline, depression and anxiety symptoms were significantly lower at follow-up, days of sick leave in the past month, and higher self-reported job performance. Over 90% of respondents claimed it helped improve their mental fitness. | 2 |
| Deady et al. (2020) | *HeadGear*  App that offers a risk assessment for mental health issues followed by a daily challenge for 30 days on topics such as coping skills, mindfulness provided in the app. | Mindfulness | RCT | To evaluate the effectiveness of the HeadGear app amongst Australian workers. | Australia | Workplace | 2275 | 40.26 (10.63), range 18-78, 74.2% male, 51.7% working in male-dominated industries, 62.2% general employees | Those assigned to the HeadGear arm had fewer depressive symptoms over the course of the trial compared to those assigned to the control. Prevalence of depression over the 12-month period was 8.0% and 3.5% for controls and HeadGear recipients, respectively, with odds of depression cases amongst the intervention group of 0.43. | 2 |
| Economides et al. (2018) | *Headspace* In app mindfulness training program consisting of 45 guided and unguided meditation sessions lasting from 10 to 20 min. | Mindfulness | RCT | To assess whether completing the first 10 sessions of Headspace positively impacts stress, affect, and irritability. | Global | Online | 160 | 27.75% 18-24yo and 27.75% 30-39yo, 58.5% female, 69.95% White, 58.5% University degree | While both interventions were effective at reducing stress associated with personal vulnerability, only the mindfulness intervention had a significant positive impact on irritability, affect, and stress resulting from external pressure. | 2 |
| Firestone et al., 2020 | *OL@-OR@ app* Regular culturally tailored tips on eating healthily, being more active, reducing stress, improving sleep, and managing weight were sent as app notifications (four to five tips per week) | Other | RCT | To determine the effect of OL@-OR@ on the wellbeing of Pasifika peoples. | New Zealand | Community | 794 | 29.9% 45+ yo, 65.5% female, 28.3% Samoan, 45.0% completed secondary school, 69.5% living in urban regions | Relative to baseline, there were significant differences between the intervention and control groups for the ‘family/community’ wellbeing, at the end of the 12-week trial. There were no significant differences observed for all other wellbeing domains for both groups. Based on our multivariate regression  analyses, education and acculturation (assimilation and marginalisation) were identified as positively strong factors associated to Pasifika ‘family and community’ wellbeing. | 4 |
| Flett et al. (2019) | *Headspace and Smiling Mind* HeadSpace includes meditations such as body scans and mindfulness. Smiling Mind offers hundreds of hours of guided and unguided mindfulness meditation practices targeting different age groups  and themes. | Mindfulness | RCT | To test the effectiveness of Headspace and Smiling Mind on mental health compared to a control app. | New Zealand | University/College | 208 | 20.08 (2.8), range 18-49, 73.6% New Zealand European | Mindfulness app users showed significant improvements in depressive symptoms, college adjustment, resilience (Smiling Mind only), and mindfulness (Headspace only) from baseline to the end of 10 days relative to control participants. Participants who continued to use the app frequently were more likely to maintain improvements in mental health, e.g. in depressive symptoms and resilience (Headspace only), until the end of the 30-day period. | 2 |
| Giraldo‐O'Meara & Doron (2020) | *GG Self Esteem* Assessments, quizzes and exercises on maladaptive beliefs linked to low self-esteem | CBT | Nonrandomised | To examine the associations between self-esteem ratings and the use of GG Self Esteem. | Global | Online | 5320 | 26.55 (10.56), range 9-88, 75.4% men, 71.7% were from countries with English as a speaking language including the US (41.4%) | Significant increases in self-esteem ratings were found across all three-time points. Increased mood ratings were only found at Level 20, compared to baseline. | 2 |
| Ha & Kim (2020) | *Spring* Users post their questions online and the answers are crowdsourced within 24 hours from counsellors. Users rate the preferred responses. | CBT | Mixed Methods | To investigate the efficacy of Spring with students in their 20s. | Korea | Online | 68 | Mean age of 21.47, 70.5% female | Depression levels were reduced significantly only in the experimental group using the app. | 3 |
| Haeger et al. (2020) | *Act Daily* Users fill out an EMA and then are offered skills sessions on topics such as identifying values and present moment acceptance. | ACT | Nonrandomised | To determine the effects of ACT Daily on mental health and wellbeing. | USA | University/College | 11 | 23.55 (5.11), range 20-38, 81% female, 91% Non-Hispanic White | Results indicated that ACT Daily was acceptable and that participants improved on depression and anxiety symptoms as well as psychological inflexibility processes over the 2 weeks. App data further indicated significant in-the-moment improvements on depression, anxiety and psychological inflexibility immediately following skill coaching, with these effects becoming larger over time. | 3 |
| Hamamura et al. (2018) | *Jibun kiroku ” [Self Record]  Users record their mood and receive psychoeducation and activities are suggested.* | CBT | Nonrandomised | To investigate effects of the Self Record App on psychological distress and alcohol consumption among Japanese workers. | Japan | Online | 557 | 38.82 (9.58), 58.8% male, 71.6% employed by a company | Results showed that continuing app users in the intervention group reported increases in anxiety, typical drinking, and heavy drinking compared to those in the control group. Negative mood regulation expectancies moderated the effects of the  intervention for general distress. | 3 |
| Hides et al. (2019) | *Music eScape* The Music eScape app analyzes each song in the users’ music library according to its level of valence and arousal and provides them with a mood map and encourages reflection on current and desired mood. | Other | RCT | To examine the effects of Music eScape app on emotion regulation, distress, and well-being. | Australia | University/College | 169 | 19.9, 79.3% female, 61.6% high school educated, 97% F/T or P/T in university ed, 85% in a relationship. | The trial found no differential improvements from app access at 1 month in emotion regulation, mental distress, or well-being. Nevertheless, improvements on 5 out of the 6 emotion regulation strategies, mental distress, and well-being were evident in both groups over the 6-month trial. | 3 |
| Howells et al. (2016) | *Headspace  Headspace* offers guided meditations that involve meditation techniques such as body scanning, guided breathing, and work on focus. | Mindfulness | RCT | To explore the viability of a positive psychological intervention. | Global | Online | 194 | 40.7 (10.6), 86.6% female, 90% White, 65.3% employed, 41.3% postgraduate-level educated, 51% married. | Results showed statistically significant increases in positive affect with a medium effect size and reduced depressive symptoms with a small effect size, although no statistically significant differences in satisfaction with life, flourishing or negative affect were found. No statistically significant gains were observed in the control condition. | 2 |
| Huberty et al. (2019) | *Calm Calm* is a consumer-based mindfulness meditation mobile app that offers a range of mindfulness meditation practice guide modules that vary in length, instruction, and content. | Mindfulness | RCT | To test the initial efficacy and sustained effects of Calm in college students with elevated stress. | USA | University/College and Online | 109 | 20.41 (2.31), 88% female, 31% freshman, 77% non-Hispanic, 59% White | The results demonstrate significant between-group differences on all main outcomes variables including perceived stress, all five factors of mindfulness (observing, describing, acting with awareness, non judgment of inner experience, and nonreactivity to inner experience), and self-compassion postintervention. These effects were sustained at follow-up, and effect sizes ranged from  moderate (0.59) to large (1.24) across all outcomes. | 4 |
| Hwang & Jo (2019) | The app-based stress management program consisted of music focused on healing, meditation, breathing methods, and yoga intervention, including health information for mental health care every week. | Combination | RCT | To assess the efficacy of a stress-management app. | Korea | Health Centre or Health Service | 60 | 53.5% 31-40yo, 94.6% female, 51.8% married. | The results indicated that stress, emotional labour, self-efficacy, and well-being were significantly different in the experimental group, but the control group’s average scores did not change significantly. However, depression and anxiety were not significantly different. | 2 |
| Kawadler et al. (2020) | *Biobase BioBase* includes workplace-specific psycho-educational content based on the job demands-resources model and combines elements of mindfulness, cognitive behavioral therapy and behavioral activation theory. | Combination | Nonrandomised | To assess changes in anxiety and well-being after the use of BioBase. | UK | Online and Workplace | 70 | Mean age of 34.67 years, 54.54% females | We found significant decreases in anxiety and increases in mental well-being after 4 weeks of using BioBase in a workplace setting. Importantly, this study found that higher baseline stress levels were associated with greater decreases in anxiety and increases in well-being. | 5 |
| Krafft et al. (2019) | *Mind Matrix* App based on recording behaviour and working with users' goals and values. | ACT | RCT | To replicate and extend initial research on a self-guided ACT intervention. | Global | University/College and Community | 98 | 22.41 (11.74), 69.35% female, 95.55% White, 91.9% Non-Hispanic/Latino | Findings indicated no differences between app conditions and a waitlist condition in the SONA credit sample. However, in the help-seeking sample, improvements were found on well-being and valued action in participants who used the app, with  greater improvements and app adoption for those using a complex version with additional skills. | 2 |
| Lee & Jung (2018) | *DeStressify* App includes mindfulness videos, text and audio delivered via a plan. | Mindfulness | RCT | To evaluate the efficacy of DeStressify among university students. | Canada | University/College | 206 | 20.6, range 16-47, 62.5% female, 68% White | Using DeStressify was shown to reduce trait anxiety and improve general health, energy, and emotional well-being in university students, and more participants in the experimental condition believed their productivity improved between baseline and postintervention measurements than the number of participants expected to believe so randomly by chance. The app did not significantly improve stress, state anxiety, physical and social functioning, and role limitations because of physical or emotional health problems or pain. | 3 |
| Levin et al. (2019) | *Act Daily*  Compared *ACT Dail*y with tailored skills to user moods, *ACT Daily* with non-tailored skills, and with a control with Ecological Momentary Assessment (EMA) but no skills offered. | ACT | RCT | To examine the impact of tailoring ACT skills based on users' assessment responses. | USA | University/College | 69 | 21.9 (5.47), range 18-46, 68.1% female, 94.2% White, 26% employed full time | Participants in the tailored app improved significantly more on psychological distress and positive mental health relative to the random app and EMA-only conditions. However, no differences were found between the random app and EMA-only conditions on outcomes. Between-group differences over time were also found on psychological inflexibility, but this appeared to be primarily due to a lower rate of improvement in the random app condition relative to both tailored and EMA-only. | 2 |
| Levin et al. (2020) | *Stop, Breathe & Think* Assesses users’ emotional state at the start of a session, to guide suggestions for mindfulness exercises. | Mindfulness | RCT | To evaluate the feasibility and acceptability of Stop, Breathe & Think amongst University students on a waitlist for counselling. | USA | University/College | 23 | 20.38 (2.43), 100% female, 91% non-Hispanic/Latino, 100% White | Very preliminary support was found for potential app efficacy relative to the control condition, particularly for depression, anxiety, and overall distress. Weaker, mixed effects were found for mindfulness and values processes. | 4 |
| Litvin and Maier (2019) | *PsycApps* Includes psychoeducation, self- assessment, journaling, self-management, and goal-setting | CBT | RCT | To evaluate the effectiveness of PsycApps. | Germany | University/College | 276 | The sample was primarily female, white, married and earning an annual salary of between $9K – 37K. | Results of repeated measures of ANOVAs showed statistically significant increases when using the app over a timeframe of four weeks. The app significantly lowered the test group’s depression levels, measured by the BDI as .57. While not significant, anxiety levels were lowered by .19 and, notably, the Life Satisfaction score was also lowered by .27. | 1 |
| Litvin et al. (2020) | *eQuoo* Includes gamification of skills, progress through a story map, new stories for each level and winning coins. | Other | RCT | To examine the impact of eQuoo on self-reported resilience and well-being. | Germany | Workplace | 709 | 28.57% 35-44 years old, 61.37% male, 87.57% White. | Repeated-measures ANOVA revealed statistically significant increases in resilience in the test group compared with both control groups over 5 weeks. The app also significantly increased personal growth, positive relations with others, and anxiety. Intervention delivered via eQuoo significantly raised mental well-being and decreased self-reported anxiety while enhancing adherence in comparison with the control conditions. | 4 |
| Ly et al. (2012) | *Shim* A conversational agent helps the user reflect upon, learn and practice CBT strategies such as present moment awareness | Combination | Nonrandomised | To examine the effectiveness of Shim on mental health and wellbeing. | Online | Online | 11 | 29.5 (5.96), range 22-42, 63% female. | The group analyses showed that the participants increased their valued action and psychological flexibility significantly during the intervention. Furthermore, value-based actions and psychological  flexibility showed small effect sizes when comparing pretest and posttest score. | 3 |
| Ly et al. (2017) | *Shim*  A fully automated conversational agent and the conversations are centred around insights, strategies and activities related to the field of positive psychology and CBT. | CBT | Mixed Methods | To assess the effectiveness and adherence of Shim. | Sweden | University/College and Online | 30 | 23.25 (7.1), range 20-49, 54% female, 64% students, 46.5% single/divorced. | Findings revealed that participants who adhered to the intervention showed significant interaction effects of group and time on psychological well-being and perceived stress compared to the wait-list control group, with small to large between effect sizes. The qualitative data revealed sub-themes which, to our knowledge, have not been found previously, such as the moderating format of the chatbot. | 4 |
| Mak et al. (2018) | *Living with Heart* This app contains 3 programs: mindfulness, self-compassion, and cognitive behavioural psychoeducation. All of them include common features such as a mood tracking function, well-being tips, a sticker earning, and a practice alarm feature. | Combination | RCT | To examine the efficacy of 3 apps in improving mental well-being and reducing psychological distress. | Hong Kong | Online | 2282 | 33.64 (12.08), 72.88% female, 79.59% received or  were receiving tertiary education (undergraduate or above). | All 3 conditions (mindfulness-based program: N=703; cognitive behavioral psychoeducation: N=753; self-compassion program:  N=705) were found to be efficacious in improving mental well-being and reducing psychological distress. All conditions enhanced mindful awareness at postprogram. Significant interaction effect was found on self-compassion; cognitive behavioral psychoeducation and self-compassion program, but not mindfulness-based program, significantly enhanced self-compassion at postprogram. | 2 |
| Mak et al. (2019) | *Living with Heart*  This compared the self-compassion vs. cognitive behavioural programme of the Living with Heart app - see above. | Combination | RCT | To compare a mobile self-compassion programme with a mobile cognitive-behavioural programme. | Hong Kong | Online | 1543 | 33.57, 73.7% female, 80.2% received tertiary education, 26.1% college students, 54% working full time. | In both groups, mental health increased at postprogramme and at 3-month follow up. Psychological distress decreased at postprogramme and at 3-month follow-up. Self-compassion and emotional regulation improved in both groups over time. | 2 |
| McCloud et al. (2020) | *Feel Stress Free* App uses CBT-based activities to help users manage symptoms of depression and anxiety. The app comprises 4 behavioral relaxation activities, one cognitive activity, a relaxing minigame, and a feature for positive messages in a bottle. | CBT | RCT | To evaluate the effectiveness of Feel Stress Free in students. | UK | University/College | 168 | 24.3 (6.71), range 18-54, 82.7% female, 61.9% undergraduate students. | At week 6, the primary end point, there was evidence that the Feel Stress Free app reduced depression symptoms but only very weak evidence that it reduced anxiety symptoms. At week 4, there was evidence to support the effectiveness of the intervention for anxiety symptoms and, though weaker, depression symptoms. | 2 |
| McEwan et al. (2019) | *Schmapped* Based on a positive psychology intervention that tasks people to notice ‘three good things’ daily, with consequent sustained improvements in wellbeing outcomes. | Other | RCT | To examine the efficacy of a nature-based intervention in improving wellbeing. | UK | Community | 582 | 28.22 (10.10), 59.8% female, 26.35% BAME. | There were statistically significant and sustained improvements in wellbeing at one-month follow-up. Importantly, in the noticing urban nature condition, compared to a built space control,  improvements in quality of life reached statistical significance for all adults and clinical significance for those classified as having a mental health difficulty. This improvement in wellbeing was partly  explained by significant increases in nature connectedness and positive affect. | 2 |
| Meinlschmidt et al. (2016) | *Smartphone Based Psychotherapeutic Micro Interventions*  The app consisted of micro-interventions during which participants practiced the psychotherapeutic techniques they had previously learned on the preliminary testing day such as viscerosensory attention, emotional imagery, facial expression, and contemplative repetition. | psychotherapy | RCT | To explore the use of smartphone-based micro interventions and related changes in mood. | Switzerland | University/College | 31 | 24.32 (2.27), range 19-29, 15.15 (1.38) years of full-time education, 74.07% single, 88.89% highest degree high school or equivalent. | Mixed models indicated that subjects showed better mood and became more awake and calmer from pre- to post-micro-intervention. These mood improvements from pre- to post-micro-intervention were associated with changes in mood from the 1st day until the last day with regard to GB mood, but not AT mood and CN mood. | 3 |
| Moberg and Beermann (2019). | *Pacifica* Mobile app marketed as a guided self-help tool for the management of stress, anxiety, and depression. | EMI | RCT | To validate the effectiveness of Pacifica. | Global | Online | 500 | 30.2 (10.85), 74.5% female, 82% White, 33.5% college diploma, 64.5% never married. | We found significant interactions between time and group. Participants in the active condition demonstrated significantly  greater decreases in depression, anxiety, and stress and increases in self-efficacy. Although we did not find a relationship between overall engagement with the app and symptom improvement, participants who completed relatively more thought record exercises sustained improvements in their symptoms through the 2-month follow-up to a greater degree than those who completed fewer. | 2 |
| Morris et al. (2010) | *Mood Sampling Application*  The application consisted of mood reporting scales and mobile therapies. | CBT | Mixed Methods | To examine the potential of apps to broaden access to CBT techniques and to provide in-the-moment support. | USA | Health Centre or Health Service | 10 | 37 (5.75), 60% women | Five case studies illustrate participants' use of the mobile phone application to increase self-awareness and to cope  with stress [...] Similar changes were observed among other participants as they used the application to negotiate bureaucratic  frustrations, work tensions and personal relationships. | 4 |
| Ponzo et al. (2020) | *BioBase* An app consisting of psychoeducational content on mental health and well-being, mood tracking (via an ecological momentary assessment, EMA), and in-the-moment exercises (e.g., deep breathing and relaxation techniques). | Combination | RCT | To test the efficacy and sustained effects of BioBase and paired wearable device (BioBeam) in university students. | UK | University/College | 262 | 19.87 (1.80), 64% female. | We found that a 4-week intervention with the BioBase program significantly reduced anxiety and increased perceived well-being, with sustained effects at a 2-week follow-up. Furthermore, a significant reduction in depression levels was found following the 4-week usage of BioBase. | 3 |
| Roy et al. (2017) | *LifeArmor, PE Coach, Positive Activity Jackpot, Eventful, Tactical Breather, Virtual Hope Box, Daily Yoga & Simply Yoga* LifeArmor and PE Coach provide psychoeducation about PTSD-related symptoms; *Positive Activity Jackpot* (Android only) or *Eventful* (iPhone only) facilitate social engagement and reduce avoidant symptoms; and *Tactical Breather* (guides use of relaxation breathing), *Virtual Hope Box* (provides multiple meditation sequences and other relaxation techniques), and *Daily Yoga* (Android) or *Simply Yoga* (iPhone) provide 20–40 minutes of yoga, all intended to reduce hyperarousal symptoms. | Combination | RCT | To investigate whether apps can reduce anxiety and depression in people with subthreshold PTSD symptoms. | USA | Health Centre or Health Service | 144 | 33.55 (11.05), 54% male, 56% non-Hispanic White. | Participants in both groups reported reductions in PTSD, anxiety, and depression symptoms during the 6-week intervention, which were sustained at 3 months, but exhibited partial rebound at 6–12 months. | 3 |
| Stallman (2019) | *My Coping Plan  A*pp is based on the transdiagnostic, strengths-focused coping planning approach to suicide prevention. The app focuses users' attention on what to do—that is, cope. | Other | RCT | To evaluate the effectiveness of My Coping Plan app in improving mental health and coping. | Australia | University/College | 56 | 28.79 (11.05), 91.1% female, 75% Psychology students, 30.4% third year students. | At 1-month follow-up, participants in the intervention condition  reported significantly lower psychological distress, improved wellbeing, and improved healthy coping strategies compared with the control condition. There was no significant difference between groups in reported unhealthy coping strategies. | 4 |
| Throuvala et al. (2020) | *Antisocial, Headspace & Pacifica* Self-monitored screen time/social media use and for voluntary self-exclusion (block app after time limit is reached). *Headspace* (mindfulness) provided brief mindfulness sessions. *Pacifica* (mood tracking) encouraged monitoring and tracking an individual’s emotional state at various times during the day to enhance awareness. | Combination | RCT | To test the efficacy of an online intervention on levels of smartphone distraction and psychological outcomes. | Global | University/College and Online | 252 | 20.72 (3.12), range 18-32, 82% female, 59.3% undergraduate Psychology students in the UK. | Results indicated high effect sizes in reduction of smartphone distraction and improvement scores on a number of self-reported secondary psychological outcomes. The intervention was not effective in reducing habitual behaviours, nomophobia, or time spent on social media. Mediation analyses demonstrated that: (i) emotional self-awareness but not mindful attention mediated the relationship between intervention effects and smartphone distraction, and (ii) online vigilance mediated the relationship between smartphone distraction and problematic social media use. | 3 |
| van Emmerik et al. (2018) | *VGZ Mindfulness Coach* App offers 40 audio exercises (e.g., yoga, body scan, attention), as well as background information on meditation and mindfulness. | Mindfulness | RCT | To investigate whether the VGZ Mindfulness Coach app can achieve improvements in wellbeing. | Global | Online | 377 | 44.71 (9.79), 96% female, 62.4% completed tertiary education, 75.3% in a relationship. | Compared to control participants, app users reported large and statistically significant increases of mindfulness after 8 weeks and small-to-medium increases of the Observing, Describing, Acting with awareness, Nonjudging, and Nonreactivity mindfulness facets as measured with the Five Facet Mindfulness Questionnaire. Also, there were large decreases of general psychiatric symptoms and moderate increases of psychological, social, and environmental quality of life. Except for social quality of life, these gains were maintained for at least 3 months. | 2 |
| Walsh et al. (2019) | *Wildflowers* App includes guided meditations and also provides didactic content in the form of lessons and information about the benefits of mindfulness training. It was designed to collect user’s ratings of current mood and stress level as well as heart rate, before and after each guided meditation session. | Mindfulness | RCT | To investigate the efficacy of Wildflowers. | Canada | University/College | 108 | 20.01 (2.53), 84% female. | Analyses revealed both state and trait effects specific to MT; participants engaging in MT demonstrated improved  mood and a reduction of stress immediately after each training session compared with before the training session  and decreased postsession stress over 3 weeks. In addition, MT relative to cognitive training resulted in greater  improvements in attentional control. Interestingly, both groups demonstrated increased subjective ratings of awareness  and acceptance from pre- to postintervention, with greater changes in acceptance for the MT group trending. | 4 |
| Weber et al. (2019) | *Kelaa Mental Resilience*  App aims to reduce stress and increase wellbeing of the user, specifically in the workplace. The app is designed to implement lifestyle changes through (1) measuring behavior, cognitions, and emotions (tracking module) and (2) providing psychoeducational content (intervention module). | Combination | RCT | To examine whether “Kelaa Mental Resilience” drives improvements in stress and wellbeing. | Global | Workplace | 678 | 40.62 (11.9), range 17-72, 75.6% female, 34.2% Bachelor degree. | The results suggest that using the mobile health intervention (vs. waitlist control group) significantly improved stress and wellbeing over time. Higher engagement in the intervention increased the beneficial effects. Additionally, use of the sleep tracking function led to an improvement in sleeping troubles. The intervention had no effects on measures of physical health or social community at work. | 1 |
| Wen et al. (2017) | *Headspace  Headspace* offers guided meditations that involve meditation techniques such as body scanning, guided breathing, and work on focus. | Mindfulness | Nonrandomised | To explore the feasibility of using Headspace as a tool to improve resident wellness. | USA | Health Centre or Health Service | 50 | Of the 30 participants included in the analysis, 90% were female. | Administration of the Headspace app for 30 days resulted in  improvement in both positive affect and mindfulness scores.  Increased usage of the app was significantly associated with increased frequency of self-reported mindfulness episodes outside the app. | 4 |
| Wylde et al. (2017) | *Headspace Headspace is a guided mindfulness meditation platform which combines deep expertise in teaching mindfulness with an approachable and bite-sized presentation.* | Mindfulness | Nonrandomised | To compare a traditionally delivered mindfulness (TDM) intervention to a smartphone delivered mindfulness (SDM) intervention. | USA | Health Centre or Health Service | 95 | 56% 23-30 years, 92% female, 52% White | Nurses in the SDM group reported significantly more “acting with awareness” and marginally more “non-reactivity to inner experience” skills compared to the TDM group. The smartphone intervention group also showed marginally more compassion satisfaction and marginally less burnout. Additionally, nurses in the SDM group had lower risk for compassion fatigue compared to the TDM group, but only when the nurses had sub-clinical posttraumatic symptoms at the start of the residency training program. | 3 |
| Yang et al. (2018) | *Headspace  Headspace* is an audio-guided mindfulness meditation program. | Mindfulness | RCT | To assess whether Headspace could decrease perceived stress and improve well-being for medical students. | USA | University/College | 88 | 25.11, range 21-47, 63.6% female, 46.6% Caucasian, 100% in medical school, 36.4% second year. | There was a significant interaction between time and treatment group for perceived stress and wellbeing. Perceived stress significantly decreased for the intervention group from T1 to T3. General well-being significantly increased for the intervention group compared to the control group from T1 to T2, and the increase was sustained through T3. | 2 |
