# Supplementary material for: Mobile Apps That Promote Emotion Regulation, Positive Mental Health, and Well-being in the General Population: Systematic Review and Meta-analysis
Source: JMIR Ment Health. 2021 Nov 8;8(11):e31170. doi: 10.2196/31170 (PMC8663676; doi:10.2196/31170)
Supplement: Multimedia Appendix 3 [file mental_v8i11e31170_app3.pdf]

An overview of the emerging evidence for MHapps

App Usage Data

Studies captured app usage or engagement data in a range of ways. Less than half of the total studies collected objective app usage data (e.g. time spent using the app or time spent using specific app features) (*k* = 23, 44%) [20,21,23,52,67,70,72,73,74,78,79,83,87,88,89,92,93,97,98,100,116,117,130]. Other studies used other measures such as participant diaries or short questionnaires to record app usage (*k* = 16, 31%) [66,69,71,76,80,81,82,85,86,90,91,101,103,105,107,129]. Some studies did not report app usage data, or it was unclear (*k* = 13, 25%) [64,65,68,75,77,84,94,95,96,99,108,113,114].

Mental Well-being

RCTs

A randomized study design was used to examine the effectiveness, acceptability, or feasibility of four MHapps targeting mental well-being outcomes across four articles [1–4]. Participants were recruited in educational settings (*k* = 2), online (*k* = 1), and from the community (*k* = 1). The methodological quality of the included studies ranged from 1 to 4 (out of a possible 5). To be eligible for inclusion in the studies, participants had to be 18 years or older, own a smartphone, have no history of mental health disorders, have regular access to the internet and an email account, and be willing to provide written consent [1–4]. Studies reported varying recruitment periods and intervention use periods up to 12 weeks [2]. A significant increase in overall [66] and family/community [66] well-being was reported after app use, compared to controls, in two randomized control trials [2,4]. Adherence to the apps varied and was measured by participation or usage (87.69%, 41.9%) or response to app notifications (significantly higher in females (M = 114.1, *SD* = 37.2) than males (M = 72.4, *SD* = 47.4)). Common limitations reported by the primary studies included the limited ability to capture which aspects of the intervention contributed to mood changes (as app user journeys were not tracked) [3,4], relatively high dropout rates without data for reasons for attrition [4], and relatively homogenous populations [2,3].

Nonrandomized Studies

A nonrandomized study design was used to examine the effectiveness of one app targeting mental well-being [5]. Researchers adopted a quasi-experimental design, obtaining pre and post-test data. Participants were recruited online. The methodological quality of the study was 2 (out of a possible 5). To be eligible for inclusion in the study participants had to be current app users. This study reported an intervention use period of at least 12 days (recommended by the researchers) rather than a strict limit. A significant increase in self-esteem and mood ratings was reported after app use. Adherence to the app was low, with Giraldo-O'Meara and Doron (2020) reporting over 80% dropout almost halfway through the levels of the intervention.

Mental Health

RCTs

A randomized study design was used to examine the effectiveness, acceptability or feasibility of 18 apps targeting mental health outcomes (e.g. anxiety, depression and stress) across 10 articles [6–15]. Participants were generally recruited in educational settings (k = 4) and online (k = 3). The methodological quality of the included studies ranged from 1 to 4. Studies reported varying recruitment periods and intervention use periods ranging from 4 weeks [13] to 6 weeks [7]. A significant decrease in mental health symptoms was generally reported after app use [13]. Adherence to apps varied from 21% to 100% across studies. More specifically, Birney et al (2016) reported participants logged into the *MoodHacker* app 16.0 times (SD= 13.3) for a total of 1.3 hours (SD= 1.3). Lower adherence rates were associated with higher anxiety and depression in Arean et al.'s (2016) study. Common limitations included participants not downloading the app after completing baseline assessments, attrition across the studies, small sample size, short duration of the intervention, and homogeneity of the sample population making generalization difficult [13].

Emotion Regulation

Mixed Methods

A mixed methods study design obtaining qualitative and quantitative data was used to examine the effectiveness of one app which targeted emotion regulation [75]. This study reported preliminary findings from pilot research as part of a field study. Participants were recruited in a clinical setting. The methodological quality of this study was 4 out of a possible 5. Health coaches provided information about the study to people that they thought might be interested in participating. The study reported an intervention use period of one month [16]. Significant increases in emotion regulation and self-awareness were reported after app use [16]. No adherence data were reported.

Integrated Outcomes

RCTs

A randomized study design was used to examine the effectiveness, acceptability or feasibility of 29 apps targeting emotional regulation, mental health and well-being outcomes across 25 articles [17,18,27–36,19,37,20–26]. Seven studies reported preliminary findings from pilot or feasibility research [20,21,26,37,38,40,41]. Participants were generally recruited in educational settings (k = 12) and online (k = 7). The methodological quality of the included studies ranged from 1 to 4. Studies reported varying recruitment periods and intervention use periods from 10 days [23] to 8 weeks [33]. A significant increase in well-being, mental health and ER outcomes was generally reported after app use, compared to controls or to other interventions [19,20,22,24,26,39,40]. Adherence to app use and retention in the research varied between 14-100%. Lower retention rates were reported for studies reporting findings from 3 and 4 month follow ups. Higher adherence rates were found in studies that reported on usage of the app at least once or those reporting a sign up or creation of an account. Adherence was generally based on completion of research outcome measures, self-report usage or software built on the backend of the app itself. Some studies reported the average number of days or number of times a specific task was completed [17,24,26,35,40,42]. Reasons for low adherence were mainly reported as ‘reasons unknown’ or failing to complete outcome measures. Common limitations included high rates of attrition, not investigating the effectiveness of apps beyond the intervention period, biased samples (e.g. self-selected or only included undergraduate students, females, or majority White participants). In some instances, the primary authors noted that the research team was not formally blinded to app allocation or the sample sizes were not sufficiently powered to identify between-group differences [29,36].

Nonrandomized Studies

A nonrandomized study design was used to examine the effectiveness, acceptability, or feasibility of nine MHapps across nine articles [18,43–49]. Researchers generally adopted a quasi-experimental design obtaining pre and post-test data. Five studies reported preliminary findings from pilot or feasibility research [34,45,48–50]. Participants were generally recruited online (*k* = 5). The methodological quality of these studies ranged from 2 to 5. Studies reported varying recruitment periods and intervention use periods from 2 weeks [48] to 5 weeks [44]. A significant increase in well-being, mental health and ER outcomes was generally reported after app use, compared to controls or to other interventions [18,43,50]. Adherence to app use and retention in the research also varied, reporting about 40-57% in alpha and beta testing [44] or about 80% in a larger study [49]. One study using a small sample (*n* = 11) of volunteers was able to retain all participants [51]. Studies also reported app use ranging from once per day over a 2-week study period to an average of 5.15 (*SD* = 6.08) times over a 4-week period [48,50]. Another study reported that paid participants used the app on more days, completed more tasks, and had a longer total duration of use than unpaid participants [45]. Reported limitations included small sample sizes or difficulty to identify which component of the studied app led to a change in outcomes [45,51]. Other studies reported issues with small sample sizes that were primarily White female participants and variation in the extent that participants used the app during the study period [48].

Mixed Methods

Mixed methods study designs were used to examine the two apps [46,52]. Following other study designs, the two studies reported preliminary findings from pilot or feasibility research. Similarly, participants were recruited in educational settings (e.g. a university). The methodological quality of these studies were 4 and 5. To be eligible for inclusion in the study participants had to be at least 18 years of age, not be in receipt of any psychological treatment including any psychopharmacological drugs — or if doing so, be on a fixed dose for more than one month —, and have continuous access to an iPhone or simply being interested in stress management and willing to use a prototype mobile app. Studies reported varying recruitment periods and intervention use periods from 2 weeks to 1 month [46,52]. A significant change in well-being and mental health outcomes (increased satisfaction with life and reduced stress but not anxiety) were generally reported after app use [46,52]. Similar to other study types, adherence to app use and retention in the research were moderate, with studies reporting 38-50% active users within 2 weeks to 1-month periods, and average session durations of about 12.3 (*SD* = 5.2, range = 4.4-24) minutes. Qualitative findings generally described themes of acceptability, benefits such as learning mindfulness, changing attitudes and barriers to usage [46,52]. Common limitations included a small sample size, a short intervention period, lack of an active control group, not using the app on participants' own phone (so deviating from naturalistic conditions), and not being able to account for confounding factors in the study [46,52].

References

1. Daugherty DA, Runyan JD, Steenbergh TA, Fratzke BJ, Fry BN, Westra E. Smartphone delivery of a hope intervention: Another way to flourish. PLoS One. Public Library of Science; 2018;13(6). PMID: 29856805
2. Firestone R, Cheng S, Dalhousie S, Hughes E, Funaki T, Henry A, et al. Exploring Pasii ka wellbeing: ndings from a large cluster randomised controlled trial of a mobile health intervention programme. NZMJ. 2020;133:1524.
3. Meinlschmidt G, Lee JH, Stalujanis E, Belardi A, Oh M, Jung EK, et al. Smartphone-based psychotherapeutic micro-interventions to improve mood in a real-world setting. Front Psychol. Frontiers Media S.A.; 2016;7(JUL).
4. van Emmerik AAP, Berings F, Lancee J. Efficacy of a Mindfulness-Based Mobile Application: a Randomized Waiting-List Controlled Trial. Mindfulness (N Y). Springer New York LLC; 2018;9(1):187–198. PMID: 29387266
5. Giraldo-O'Meara M, Doron G. Can self-esteem be improved using short daily training on mobile applications? Examining real world data of GG Self-esteem users. Clin Psychol. Wiley-Blackwell Publishing Ltd; 2020;
6. Carissoli C, Villani D, Riva G. Does a meditation protocol supported by a mobile application help people reduce stress? suggestions from a controlled pragmatic trial. Cyberpsychology, Behav Soc Netw. Mary Ann Liebert Inc.; 2015;18(1):46–53. PMID: 25584730
7. Birney AJ, Gunn R, Russell JK, Ary D V. Moodhacker mobile web app with email for adults to self-manage mild-to-moderate depression: Randomized controlled trial. JMIR mHealth uHealth. JMIR Publications Inc.; 2016;4(1).
8. Bruehlman-Senecal E, Hook CJ, Pfeifer JH, FitzGerald C, Davis B, Delucchi KL, et al. Smartphone App to Address Loneliness Among College Students: Pilot Randomized Controlled Trial. JMIR Ment Heal. JMIR Publications; 2020;7(10):e21496–e21496.
9. Roy MJ, Costanzo ME, Highland KB, Olsen C, Clayborne D, Law W. An App a Day Keeps the Doctor Away: Guided Education and Training via Smartphones in Subthreshold Post Traumatic Stress Disorder. Cyberpsychology, Behav Soc Netw. Mary Ann Liebert Inc.; 2017;20(8):470–478. PMID: 28737954
10. Krafft J, Potts S, Schoendorff B, Levin ME. A Randomized Controlled Trial of Multiple Versions of an Acceptance and Commitment Therapy Matrix App for Well-Being. Behav Modif. SAGE Publications Inc; 2017;43(2):246–272.
11. Arean PA, Hallgren KA, Jordan JT, Gazzaley A, Atkins DC, Heagerty PJ, et al. The use and effectiveness of mobile apps for depression: Results from a fully remote clinical trial. J Med Internet Res. JMIR Publications Inc.; 2016;18(12). PMID: 27998876
12. McCloud T, Jones R, Lewis G, Bell V, Tsakanikos E. Effectiveness of a Mobile App Intervention for Anxiety and Depression Symptoms in University Students: Randomized Controlled Trial. JMIR mHealth uHealth. JMIR Publications; 2020;8(7):e15418–e15418.
13. Levin ME, Haeger J, Cruz RA. Tailoring Acceptance and Commitment Therapy Skill Coaching in the Moment Through Smartphones: Results from a Randomized Controlled Trial. Mindfulness (N Y). Springer New York LLC; 2019;10(4):689–699.
14. Borjalilu S, Ali Mazaheri M, Talebpour A. Effectiveness of mindfulness-based stress

management in the mental health of Iranian university students: A comparison of blended therapy, face-to-face sessions, and mHealth app (Aramgar). Iran J Psychiatry Behav Sci. Kowsar Medical Publishing Company; 2019;13(2).
15. Ha SW, Kim J. Designing a Scalable, Accessible, and Effective Mobile App Based Solution for Common Mental Health Problems. Int J Hum Comput Interact. Taylor and Francis Inc.; 2020;36(14):1354–1367.
16. Morris ME, Kathawala Q, Leen TK, Gorenstein EE, Guilak F, Labhard M, et al. Mobile Therapy: Case Study Evaluations of a Cell Phone Application for Emotional Self-Awareness. J Med Internet Res. 2010;12(2):e10.
17. Bostock S, Crosswell AD, Prather AA, Steptoe A. Mindfulness on-the-go: Effects of a mindfulness meditation app on work stress and well-being. J Occup Health Psychol. American Psychological Association Inc.; 2019;24(1):127–138. PMID: 29723001
18. Bakker D, Rickard N. Engagement with a cognitive behavioural therapy mobile phone app predicts changes in mental health and wellbeing: MoodMission. Aust Psychol. John Wiley and Sons Ltd; 2019;54(4):245–260.
19. Moberg C, Niles A, Beermann D. Guided self-help works: Randomized waitlist controlled trial of Pacifica, a mobile app integrating cognitive behavioral therapy and mindfulness for stress, anxiety, and depression. J Med Internet Res. JMIR Publications Inc.; 2019;21(6). PMID: 31199319
20. Ponzo S, Morelli D, Kawadler JM, Hemmings NR, Bird G, Plans D. Efficacy of the digital therapeutic mobile app biobase to reduce stress and improve mental well-being among university students: Randomized controlled trial. JMIR mHealth uHealth. JMIR Publications Inc.; 2020;8(4). PMID: 31926063
21. Yang E, Schamber E, Meyer RML, Gold JI. Happier Healers: Randomized Controlled Trial of Mobile Mindfulness for Stress Management. J Altern Complement Med. Mary Ann Liebert Inc.; 2018;24(5):505–513. PMID: 29420050
22. Stallman HM. Efficacy of the My Coping Plan mobile application in reducing distress: A randomised controlled trial. Clin Psychol. Wiley-Blackwell Publishing Ltd; 2019;23(3):206–212.
23. Throuvala MA, Griffiths MD, Rennoldson M, Kuss DJ. Mind over matter: Testing the efficacy of an online randomized controlled trial to reduce distraction from smartphone use. Int J Environ Res Public Health. MDPI AG; 2020;17(13):1–30. PMID: 32635650
24. Walsh KM, Saab BJ, Farb NA. Effects of a Mindfulness Meditation App on Subjective Well-Being: Active Randomized Controlled Trial and Experience Sampling Study. JMIR Ment Heal. JMIR Publications Inc.; 2019;6(1):e10844.
25. Weber S, Lorenz C, Hemmings N. Improving Stress and Positive Mental Health at Work via an App-Based Intervention: A Large-Scale Multi-Center Randomized Control Trial. Front Psychol. Frontiers Media S.A.; 2019;10.
26. McEwan K, Richardson M, Sheffield D, Ferguson FJ, Brindley P. A smartphone app for improving mental health through connecting with urban nature. Int J Environ Res Public Health. MDPI AG; 2019;16(18). PMID: 31547286
27. Howells A, Ivtzan I, Eiroa-Orosa FJ. Putting the ‘app’ in Happiness: A Randomised Controlled Trial of a Smartphone-Based Mindfulness Intervention to Enhance Wellbeing. J Happiness Stud. Springer Netherlands; 2016;17(1):163–185.
28. Hides L, Dingle G, Quinn C, Stoyanov SR, Zelenko O, Tjondronegoro D, et al. Efficacy and Outcomes of a Music-Based Emotion Regulation Mobile App in Distressed Young People: Randomized Controlled Trial. JMIR Mhealth Uhealth. 2019;7(1):e11482.
29. Champion L, Economides M, Chandler C. The efficacy of a brief app-based mindfulness intervention on psychosocial outcomes in healthy adults: A pilot

randomised controlled trial. PLoS One. Public Library of Science; 2018;13(12). PMID: 30596696
30. Mak WW, Tong AC, Yip SY, Lui WW, Chio FH, Chan AT, et al. Efficacy and Moderation of Mobile App-Based Programs for Mindfulness-Based Training, Self-Compassion Training, and Cognitive Behavioral Psychoeducation on Mental Health: Randomized Controlled Noninferiority Trial. JMIR Ment Heal. JMIR Publications; 2018;5(4):e60–e60.
31. Litvin S, Saunders R, Maier MA, Lüttke S. Gamification as an approach to improve resilience and reduce attrition in mobile mental health interventions: A randomized controlled trial. PLoS One. Public Library of Science; 2020;15(9 September 2020). PMID: 32877425
32. Bakker D, Kazantzis N, Rickwood D, Rickard N. A randomized controlled trial of three smartphone apps for enhancing public mental health. Behav Res Ther. Elsevier Ltd; 2018;109:75–83. PMID: 30125790
33. Coelhoso CC, Tobo PR, Lacerda SS, Lima AH, Barrichello CRC, Amaro E, et al. A new mental health mobile app for well-being and stress reduction in working women: Randomized controlled trial. J Med Internet Res. JMIR Publications Inc.; 2019;21(11). PMID: 31697244
34. Deady M, Glozier N, Calvo R, Johnston D, MacKinnon A, Milne D, et al. Preventing depression using a smartphone app: A randomized controlled trial. Psychol Med. Cambridge University Press; 2020;
35. Economides M, Martman J, Bell MJ, Sanderson B. Improvements in Stress, Affect, and Irritability Following Brief Use of a Mindfulness-based Smartphone App: A Randomized Controlled Trial. Mindfulness (N Y). Springer New York LLC; 2018;9(5):1584–1593.
36. Flett JAM, Hayne H, Riordan BC, Thompson LM, Conner TS. Mobile Mindfulness Meditation: a Randomised Controlled Trial of the Effect of Two Popular Apps on Mental Health. Mindfulness (N Y). Springer New York LLC; 2019;10(5):863–876.
37. Lee RA, Jung ME. Evaluation of an mhealth app (destressify) on university students' mental health: Pilot trial. J Med Internet Res. JMIR Publications Inc.; 2018;20(1).
38. Levin ME, Hicks ET, Krafft J. Pilot evaluation of the stop, breathe & think mindfulness app for student clients on a college counseling center waitlist. J Am Coll Heal. Routledge; 2020;
39. Mak W, Wong C, Chan A, Lau J. Mobile self-compassion programme for promotion of public mental health: a randomised controlled trial [Internet]. Hong Kong Med J. 2019.
40. Huberty J, Green J, Glissmann C, Larkey L, Puzia M, Lee C. Efficacy of the Mindfulness Meditation Mobile App "Calm" to Reduce Stress Among College Students: Randomized Controlled Trial. JMIR Mhealth Uhealth. 2019;7(6):e14273.
41. Hwang WJ, Jo HH. Evaluation of the effectiveness of mobile app-based stress-management program: A randomized controlled trial. Int J Environ Res Public Health. MDPI AG; 2019;16(21). PMID: 31684169
42. Litvin S, Maier MA. How mHealth programmes can treat depression: A randomised controlled trial.
43. Bakker D, Rickard N. Engagement in mobile phone app for self-monitoring of emotional wellbeing predicts changes in mental health: MoodPrism. J Affect Disord. Elsevier B.V.; 2018;227:432–442. PMID: 29154165
44. Deady M, Johnston D, Milne D, Glozier N, Peters D, Calvo R, et al. Preliminary effectiveness of a smartphone app to reduce depressive symptoms in the workplace: feasibility and acceptability study. JMIR mHealth uHealth. JMIR Publications Inc.;

2018;6(12).
45. Kawadler JM, Hemmings NR, Ponzo S, Morelli D, Bird G, Plans D. Effectiveness of a Smartphone App (BioBase) for Reducing Anxiety and Increasing Mental Well-Being: Pilot Feasibility and Acceptability Study. JMIR Form Res. 2020;4(11):e18067.
46. Ly KH, Ly AM, Andersson G. A fully automated conversational agent for promoting mental well-being: A pilot RCT using mixed methods. Internet Interv. Elsevier B.V.; 2017;10:39–46.
47. Morrison Wylde C, Mahrer NE, Meyer RML, Gold JI. Mindfulness for Novice Pediatric Nurses: Smartphone Application Versus Traditional Intervention. J Pediatr Nurs. 2017;36:205–212.
48. Haeger JA, Davis CH, Levin ME. Utilizing ACT daily as a self-guided app for clients waiting for services at a college counseling center: A pilot study. J Am Coll Heal. Routledge; 2020;
49. Hamamura T, Suganuma S, Ueda M, Mearns J, Shimoyama H. Standalone effects of a cognitive behavioral intervention using a mobile phone app on psychological distress and alcohol consumption among Japanese workers: Pilot nonrandomized controlled trial. J Med Internet Res. JMIR Publications Inc.; 2018;20(3).
50. Wen L, Sweeney TE, Welton L, Trockel M, Katznelson L. Encouraging Mindfulness in Medical House Staff via Smartphone App: A Pilot Study. Acad Psychiatry. 2017;41(5):646–650.
51. Ly KH, Dahl JA, Carlbring P, Andersson G. Development and initial evaluation of a smartphone application based on acceptance and commitment therapy. Springerplus. SpringerOpen; 2012;1(1):1–11.
52. Ahtinen A, Mattila E, Välkkynen P, Kaipainen K, Vanhala T, Ermes M, et al. Mobile Mental Wellness Training for Stress Management: Feasibility and Design Implications Based on a One-Month Field Study. JMIR mhealth uhealth. JMIR Publications Inc.; 2013;1(2):e11.
