# Supplementary material for: Mobile Apps That Promote Emotion Regulation, Positive Mental Health, and Well-being in the General Population: Systematic Review and Meta-analysis
Source: JMIR Ment Health. 2021 Nov 8;8(11):e31170. doi: 10.2196/31170 (PMC8663676; doi:10.2196/31170)
Supplement: Multimedia Appendix 4 [file mental_v8i11e31170_app4.pdf]

## An overview of the theoretical underpinning of the reviewed MHapps

### Mindfulness

Mindfulness was a common theoretical approach applied in the MHapps (18/52; 25%). The MHapps that were informed by a mindfulness approach (e.g., *Headspace, Calm, It's Time to Relax*) offered users spoken meditations and meditation training during a specified programme of regular practice. Meditations took various forms, such as encouraging users to distance themselves from thoughts and emotions, and connect with their body via techniques such as a Body Scan (participants bring mindful awareness to different parts of the body), visualisation, or developing awareness of emotions (e.g., *Mindfulness-Based Resilience Training app, DeStressify, Aramgar*) [1,2].

### CBT

CBT was another common approach applied in 10 studies (19% of the reviewed articles). CBT formed the basis of psychoeducation provided to users in apps to promote well-being (e.g., *Spring, Jibun kiroku, Shim, Living with Heart)* and manage symptoms of disorders such as anxiety and depression *(e.g., Feel Stress Free).* Some CBT apps aimed to help users identify factors associated with a change in mood or emotion, identify "automatic thoughts", adopt new thinking styles and encourage users to use cognitive reappraisal to manage stressful situations. Other CBT concepts included becoming aware of self-critical beliefs, tolerating uncertainty, avoiding *globalizing* (generalising from specific instances), challenging maladaptive beliefs, and adopting positive coping strategies, such as in the *GG Self Esteem* app [3]. Core concepts from CBT were generally taught as part of modules in psychoeducation (e.g., *Equoo*) [4].

### ACT

ACT principles were applied in 4 studies (8% of MHapps) (e.g., *Act Daily, Mind Matrix*) [5,6]. MHapps applied insights from the ACT model such as *acceptance* (willingness to experience difficult emotions and experiences directly), *defusion* (relating to thoughts with reduced emotion), *present moment awareness* (flexible attention to current experiences), and identification of *values* (aspects of personal meaning and import) [7]. A key goal in ACT was to promote flexibility in behaviour based on personally-held values or direct contingencies [8].

### EMA & EMI

Ecological Momentary Assessment (EMA) referred to the sampling of users' experiences in real time [9]. Relatedly, Ecological Momentary Intervention (EMI) referred to intervening in the users' life in the context of their everyday lives [10]. EMA and EMI were combined with other theoretical approaches. For instance, in one study, EMA was combined with CBT and ACT to sample users' moods and then provide them with psychological interventions, such as CBT-based exercises [11].

Another paper reported use of EMI to provide notifications at random, such as messages of hope, with the aim of increasing the users' daily experience [10].

**Combination of multiple theoretical approaches**

Many MHapps drew on a combination of various combined theoretical approaches (11/52; 21%). In such cases, a range of tools and techniques were used, such as encouraging app users to notice positive and negative cognitions and accept negative emotions. In one app, ACT was combined with tenets of positive psychology and CBT to provide users with guidance to engage in positive activities, practice gratitude and reflect on positive experiences (*Shim*) [12]. In a number of studies, MHapps incorporated principles of ACT combined with Ecological Momentary Assessment (EMA) (e.g., *Act Daily*). This involved recording users' moods and behaviours in real time, and then the app providing users with a message to encourage acceptance of a given emotion [13].

**Other approaches**

Other theoretical approaches applied in MHapps included Behavioural Activation (BA), a psychotherapeutic approach that promoted adaptive activities with the aim of reducing the risk of developing depression. Specifically, some apps built on theories that exposure to, and observing environments within nature and experiencing gratitude, could reduce stress and increase mental focus [14]. Less commonly applied theories included the notion that planning positive activities contributed to increased subjective well-being and that social activities could reduce the risk of mental health disorders (e.g. *Positive Activity Jackpot* [15]). Another example included undertaking social challenges to increase social connections with the aim of decreasing loneliness [16].

**References:**


1. Lee RA, Jung ME. Evaluation of an mhealth app (destressify) on university students' mental health: Pilot trial. J Med Internet Res. JMIR Publications Inc.; 2018;20(1).
2. Borjalilu S, Ali Mazaheri M, Talebpour A. Effectiveness of mindfulness-based stress management in the mental health of Iranian university students: A comparison of blended therapy, face-to-face sessions, and mHealth app (Aramgar). Iran J Psychiatry Behav Sci. Kowsar Medical Publishing Company; 2019;13(2).
3. Giraldo-O'Meara M, Doron G. Can self-esteem be improved using short daily training on mobile applications? Examining real world data of GG Self-esteem users. Clin Psychol. Wiley-Blackwell Publishing Ltd; 2020;
4. Litvin S, Maier MA. How mHealth programmes can treat depression: A randomised controlled trial.
5. Haeger JA, Davis CH, Levin ME. Utilizing ACT daily as a self-guided app for clients waiting for services at a college counseling center: A pilot study. J Am Coll Heal. Routledge; 2020;
6. Krafft J, Potts S, Schoendorff B, Levin ME. A Randomized Controlled Trial of Multiple Versions of an Acceptance and Commitment Therapy Matrix App for Well-Being. Behav Modif. SAGE Publications Inc; 2017;43(2):246–272.
7. Bai Z, Luo S, Zhang L, Wu S, Chi I. Acceptance and Commitment Therapy (ACT) to reduce depression: A systematic review and meta-analysis. J Affect Disord. 2020;260:728–737.
8. Kashdan TB, Rottenberg J. Psychological flexibility as a fundamental aspect of health. Clin Psychol Rev. 2010;30(4):865–878. PMID: 21151705
9. Shiffman S, Stone AA, Hufford MR. Ecological Momentary Assessment. Annu Rev Clin Psychol. Annual Reviews; 2008;4(1):1–32.
10. Daugherty DA, Runyan JD, Steenbergh TA, Fratzke BJ, Fry BN, Westra E. Smartphone delivery of a hope intervention: Another way to flourish. PLoS One. Public Library of Science; 2018;13(6). PMID: 29856805
11. Ponzo S, Morelli D, Kawadler JM, Hemmings NR, Bird G, Plans D. Efficacy of the digital therapeutic mobile app biobase to reduce stress and improve mental well-being among university students: Randomized controlled trial. JMIR mHealth uHealth. JMIR Publications Inc.; 2020;8(4). PMID: 31926063
12. Ly KH, Ly AM, Andersson G. A fully automated conversational agent for promoting mental well-being: A pilot RCT using mixed methods. Internet Interv. Elsevier B.V.; 2017;10:39–46.
13. Levin ME, Haeger J, Cruz RA. Tailoring Acceptance and Commitment Therapy Skill Coaching in the Moment Through Smartphones: Results from a Randomized Controlled Trial. Mindfulness (N Y). Springer New York LLC; 2019;10(4):689–699.

14. McEwan K, Richardson M, Sheffield D, Ferguson FJ, Brindley P. A smartphone app for improving mental health through connecting with urban nature. Int J Environ Res Public Health. MDPI AG; 2019;16(18). PMID: 31547286
15. Roy MJ, Costanzo ME, Highland KB, Olsen C, Clayborne D, Law W. An App a Day Keeps the Doctor Away: Guided Education and Training via Smartphones in Subthreshold Post Traumatic Stress Disorder. Cyberpsychology, Behav Soc Netw. Mary Ann Liebert Inc.; 2017;20(8):470–478. PMID: 28737954
16. Bruehlman-Senecal E, Hook CJ, Pfeifer JH, FitzGerald C, Davis B, Delucchi KL, et al. Smartphone App to Address Loneliness Among College Students: Pilot Randomized Controlled Trial. JMIR Ment Heal. JMIR Publications; 2020;7(10):e21496–e21496.
