# Supplementary material for: Mobile Apps That Promote Emotion Regulation, Positive Mental Health, and Well-being in the General Population: Systematic Review and Meta-analysis
Source: JMIR Ment Health. 2021 Nov 8;8(11):e31170. doi: 10.2196/31170 (PMC8663676; doi:10.2196/31170)
Supplement: Multimedia Appendix 5 [file mental_v8i11e31170_app5.docx]

### Appendix 5

### Risk of Bias Summary: Authors’ Judgments about Risk of Bias Domains for Each Included Study

For the overall score, low ROB required random sequencing, allocation concealment, and blinding in order to be scored low risk with no other important concerns; unclear ROB was assigned if 1 or 2 domains were scored not clear or not described in the text; high ROB was assigned if >2 domains were scored not clear or not done.
