# Supplementary material for: Mobile Apps That Promote Emotion Regulation, Positive Mental Health, and Well-being in the General Population: Systematic Review and Meta-analysis
Source: JMIR Ment Health. 2021 Nov 8;8(11):e31170. doi: 10.2196/31170 (PMC8663676; doi:10.2196/31170)
Supplement: Multimedia Appendix 6 [file mental_v8i11e31170_app6.docx]

Appendix 6 Funnel plots

**Figure S1**

Funnel plot Mental Health

**Figure S2**

Funnel Plot Trim and Fill for Mental Health

**Figure S3**

Funnel plot Mental Wellbeing

**Figure S4**

Funnel plot Emotion Regulation
